# Tunable Electronic Properties of Lateral Monolayer Transition Metal Dichalcogenide Superlattice Nanoribbons

**DOI:** 10.3390/nano11020534

**Published:** 2021-02-19

**Authors:** Jinhua Wang, Gyaneshwar P. Srivastava

**Affiliations:** 1 School of Science, Tianjin University of Technology and Education, Tianjin 300222, China; 2 School of Physics, University of Exeter, Exeter EX4 4QL, UK; g.p.srivastava@exeter.ac.uk

**Keywords:** electronic properties, first-principle, edge passivation, vacancy, biaxial strain

## Abstract

The structural stability and structural and electronic properties of lateral monolayer transition metal chalcogenide superlattice zigzag and armchair nanoribbons have been studied by employing a first-principles method based on the density functional theory. The main focus is to study the effects of varying the width and periodicity of nanoribbon, varying cationic and anionic elements of superlattice parent compounds, biaxial strain, and nanoribbon edge passivation with different elements. The band gap opens up when the (MoS_2_)_3_/(WS_2_)_3_ and (MoS_2_)_3_/(MoTe_2_)_3_ armchair nanoribbons are passivated by H, S and O atoms. The H and O co-passivated (MoS_2_)_3_/(WS_2_)_3_ armchair nanoribbon exhibits higher energy band gap. The band gap with the edge S vacancy connecting to the W atom is much smaller than the S vacancy connecting to the Mo atom. Small band gaps are obtained for both edge and inside Mo vacancies. There is a clear difference in the band gap states between inside and edge Mo vacancies for symmetric nanoribbon structure, while there is only a slight difference for asymmetric structure. The electronic orbitals of atoms around Mo vacancy play an important role in determining the valence band maximum, conduction band minimum, and impurity level in the band gap.

## 1. Introduction

Properties of two-dimensional (2D) materials have been the focus of a great deal of experimental and theoretical research over the past few decades due to their unique electronic structure. 2D materials such as graphene, silicene, boron nitride, phosphorene, transition metal chalcogenides (MoS_2_, WS_2_, MoTe_2_, WTe_2_), etc., show extraordinary mechanical, electronic and optical properties which make them suitable candidates for future optoelectronic and thermoelectric applications different from bulk materials [1,2,3,4,5,6,7,8,9]. Apart from the unique properties of these materials, there are also important areas of emphasis, and aspects that have attracted extensive attention in the academic field, especially transition metal dichalcogenides (TMDCs) materials, which may be important for transport measurements and applications [6,10,11,12].

Due to the cross-plane quantum confinement effect, low dimensional monolayer TMDCs has different properties from their bulk materials. Bulk MoS_2_, WS_2_, MoTe_2_, MoSe_2_, WSe_2_, etc., are indirect band gap semiconductors, while their monolayer changes into a direct band gap semiconductor [12,13,14,15,16]. Gusakova et al. reported that monolayer MoS_2_, MoSe_2_, WS_2_, and WSe_2_ have direct band gaps of 1.88 eV, 1.57 eV, 2.03 eV, 1.67 eV respectively, and the corresponding bulk have indirect band gaps of 1.23 eV, 1.09 eV, 1.32 eV, and 1.21 eV respectively [9]. Heterojunctions, such as superlattices, have new and unique electronic and transport properties which can be applied in nanodevices. Recently, dislocation free MoS_2_-MoSe_2_, WS_2_-WSe_2_, WS_2_-MoSe_2_ and TMDCs/graphene heterostructures and superlattices were synthesized and studied [17,18,19,20,21,22]. Xie et al. successfully reported the fabrication of coherent monolayer TMDCs superlattices with precisely controlled supercell dimensions and lattice coherence maintained over the entire structures [20]. From theoretical calculations, Ding et al. found that MoS_2_-MoSe_2_ lateral superlattices show low lattice thermal conductivity compared to the individual single-layer [21]. In contrast to the isolated graphene and monolayer MoS_2_, Graphene/MoS_2_ superlattices exhibit metallic electronic properties [22]. 

The electronic band gap in TMDCs plays an important role in optoelectronic, spintronic and sensor applications. It can be adjusted through strain, electric field, doping, edge passivation to realize new applications. TMDCs nanoribbons can be fabricated by using electrochemical methods and they exhibit different electronic, transport and magnetic properties depending on the edge structure and width [8,23,24,25,26,27,28,29,30,31]. Nanoribbons can exist as zigzag and armchair based on their chirality and edge orientation. Previous research shows MoS_2_, WS_2_, MoSe_2_, WSe_2_ zigzag nanoribbons as metallic and ferromagnetic and the armchair nanoribbons as semiconducting [26,27,28,29,30,31,32]. The edges of MoS_2_ nanostructures are known to be very catalytically active [33]. Parija et al. demonstrated that edge corrugations yield distinctive spectroscopic signatures using first-principles calculations, X-ray absorption spectroscopy, scanning transmission X-ray microscopy (STXM) imaging [33]. The electronic properties of armchair MoS_2_ nanoribbon can be adjusted by passivating atoms on the edge and by changing the width, which is expected to make them useful for semiconductor functional electronic devices [24,32,34,35,36]. First-principles calculations show that armchair MoS_2_ nanoribbons passivated with H and O atoms are more stable and exhibit a direct band gap of about 1.43 eV [32]. Application of strain can tune the electronic properties of materials. Some research has been carried out on the electronic properties of TDMC monolayer and nanoribbon [15,28,37,38,39,40,41,42,43,44,45,46,47]. 

Although TMDCs themselves exhibit many unique characteristics, making them powerful candidates for future electronics and sensors, heterostructures or superlattice composed of TMDCs can further achieve the novel electronic properties that the individual materials do not exhibit alone. Given that experimental techniques are available to synthesize (a) TMDCs lateral superlattice [20] and (b) TMDCs nanoribbons [23], it is expected that TMDCs superlattice nanoribbons can also be synthesized. Such a system should offer a richer variety of electronic structure due to the presence of either two cations (such as in MoS_2_/WS_2_) or two anions (such as in MoS_2_/MoTe_2_) at ribbon edges. 

In this paper, we apply a first-principles method to investigate the structural and electronic properties of lateral monolayer transition metal chalcogenide superlattice nanoribbons. We consider the geometry, edge atom modification and vacancy effect of symmetric and asymmetric armchair (MoS_2_)_3_/(WS_2_)_3_ and (MoS_2_)_3_/(MoTe_2_)_3_ nanoribbons. The H-, H-S- and H-O-saturated nanoribbons were studied. The results show that the band gap opens up when these armchair nanoribbons (ANR) are passivated by H, S and O atoms. Especially, the (MoS_2_)_3_/(WS_2_)_3_ ANR-H-O exhibits remarkable large band gap. The effects of increasing the ribbon width and superlattice period and of biaxial strain on the band gap for the armchair nanoribbons have also been studied, which can provide insight and information for applications in electronic devices.

## 2. Method

In this work we employ the density functional theory (DFT) based plane-wave pseudopotential first-principles method as implemented in the Quantum-Espresso (QE) computer package [48,49]. The ion-electron interaction was modeled by using norm-conserving pseudopotentials, except for the W atom, for which an ultrasoft pseudopotential was used [50]. The electron exchange-correlation energy is calculated using the generalized gradient approximation as outlined by Perdew, Burke, and Ernzerhof [51]. The cutoff energy for the plane-wave basis was 60 Ry. The Brillouin zone summation was carried out using the 8 × 8 × 1 *k*-points grid mesh for the MoS_2_ monolayer. A 12 × 1 × 1 *k*-points grid mesh is used for zigzag nanoribbons and a 8 × 1 × 1 *k*-points grid mesh is used for armchair nanoribbons. Within the periodic geometry construction, the vacuum space was considered to be more than 12 Å in order to avoid the interaction between neighboring nanoribbons. Atomic geometry optimization was carried out using the BFGS (Broyden-Fletcher-Goldfarb-Shanno) algorithm as implemented in the Quantum Espresso package. 

It is well known that DFT, whether within the local density approximation (LDA) or a generalized gradient approximation (GGA), underestimates the electronic band gap of semiconductors. Use of hybrid functionals and/or a quasi-particle theory is required to obtain accurate band gap results. While these considerations are beyond the scope of the present study, it is important to point out that our consistent use of PBE-GGA will provide a consistent set of band gap results across all the structures considered in this work.

## 3. Results and Discussion

We will present band structure results with the energy zero set at the Fermi level (*E*_F_) for metallic systems and at the top of the valence band (*E*_V_) for semiconducting systems.

### 3.1. MoS_2_/WS_2_ System

#### 3.1.1. (MoS_2_)_3_/(WS_2_)_3_ Lateral Superlattice

**Figure 1:**
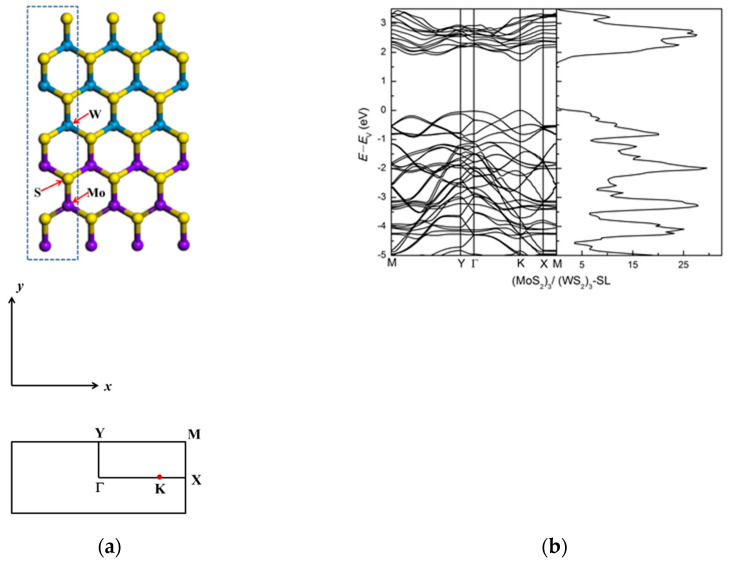
(**a**) The atomic structure of the lateral (MoS_2_)_3_/(WS_2_)_3_ superlattice. The blue rectangle shows the repeat unit cell and the high-symmetry points in Brillouin zone are marked, (**b**) The electronic band structure and density of states (dos) of the superlattice. Purple ball represents Mo atoms, blue ball represents W atoms, yellow ball represents S atoms.

We modeled a lateral superlattice (SL) as an artificially periodic system, with a unit cell normal to the monolayer containing a vacuum region of 12 Å. We first optimized the MoS_2_ and WS_2_ monolayer systems, obtaining the equilibrium lattice constant of 3.186 Å for both. Our calculated direct bandgap of 1.71 eV for MoS_2_ and 1.82 eV for WS_2_ are in good agreement with reported experimental and theoretical results [12,15,26,32,34,52,53]. The similarity of lattice parameters and different band gaps is suitable to build superlattices. We focus on (MoS_2_)_3_/(WS_2_)_3_ lateral superlattice (SL), as shown in Figure 1a. The band structure, shown in Figure 1b, indicates that this superlattice is also semiconducting and has direct band gap close to that of monolayer MoS_2_: the band gap is 1.71 eV for the (MoS_2_)_3_/(WS_2_)_3_ lateral superlattice, with both the valence band maximum (VBM) and conduction band minimum (CBM) located at the high symmetry point K of the monolayer system. The energy bands close to VBM and CBM are relatively flat along ΓY and dispersive along ΓX. The states near the band gap at the high symmetry point K are mainly derived from the *d* orbital of Mo atom and the *p* orbital of S atom.

#### 3.1.2. Zigzag (MoS_2_)_3_/(WS_2_)_3_ Lateral Nanoribbons

**Figure 2:**
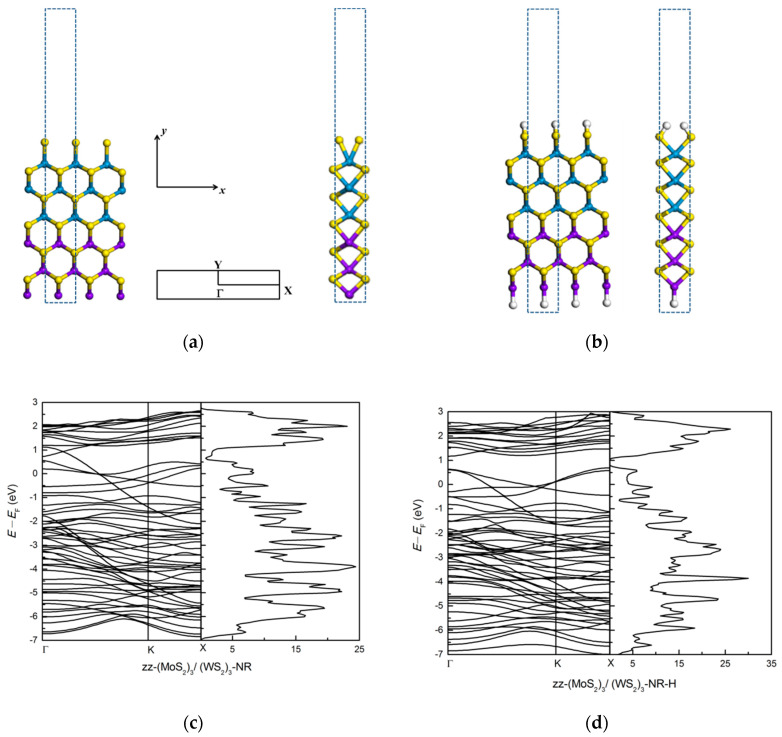
The atomic structure of zz-(MoS_2_)_3_/(WS_2_)_3_-NR and H-passivated of zz-(MoS_2_)_3_/(WS_2_)_3_ -NR are shown in (**a**,**b**), respectively. The blue dotted rectangle shows the repeat unit cell in (**a**) and the high-symmetry points in Brillouin zone are marked. In panels (**c**,**d**) we show the electronic band structure and density of states (dos) of bare and H-passivated systems, respectively.

Nanoribbons were constructed by cutting the lateral (MoS_2_)_3_/(WS_2_)_3_ monolayer superlattice. There are zigzag (zz-NR) and armchair (ac-NR) nanoribbons with different chiralities and edge structures. As shown in Figure 2a, for zz-NR we used the rectangular unit as repeat unit cell of length a along *x* direction and (3$\sqrt{3}a$ plus a vacuum region of 12 Å) along *y* direction, where a is the lattice constant. There are 3 Mo, 3 W, 6 S atoms in the repeat unit. 

The structural stability of a bare nanoribbon was examined by evaluating the edge energy per interface unit cell using the formula

*E*_edge_ = (*E*_ribbon_ − *n*_1_ × *E*_unit_(MoS_2_) − *n*_2_ × *E*_unit_(WS_2_) − *n*_Mo_ × *E*(Mo) − *n*_W_ × *E*(W) − *n*_S_ × *E*(S))/2*L* (1)

Here *E*_ribbon_ is the total energy of the nanoribbon unit cell, *E*_unit_(MoS_2_) is the total energy of bulk MoS_2_ monolayer, *E*_unit_(WS_2_) is the total energy of bulk WS_2_ monolayer, *E*(Mo) is is the total energy of bcc bulk Mo, *E*(W) is the total energy of bcc bulk W, we have considered *E*(S) as half of the total energy of a S_2_ molecule, *n*_1_ is the number of bulk MoS_2_ monolayers, *n*_2_ is the number of bulk WS_2_, *n*_Mo_ is the number of edge Mo atoms, *n*_W_ is the number of edge W atoms, and *n*_S_ is the number of edge S atoms, and *L* is the number of edge unit cells. For the zz-NR in Figure 2a the calculated edge energy is 0.89 eV. This result indicates that such a nanoribbon, terminated on one side by the metallic atom Mo and on the other side by the non-metallic atom S, is not a stable structure. Other zz-NR geometries can be constructed, as explained in [32], but we have not studied them all here. The study in [32] asserts that only S-terminated MoS_2_ zz-NR are stable geometries.

We also estimated the ‘’interface energy per interface unit cell’’ between the constituent materials of the superlattice nanoribbon (e.g., between MoS_2_ and WS_2_) by using the formula

*E*_IF_ = *E*_edge_ − (*E*_ribbon_ − *n*_MoS2_ × *E*_unit_(MoS_2_) − *n*_WS2_ × *E*_unit_(WS_2_))/2*L* (2)

where *n*_MoS2_ and *n*_WS2_ are the numbers of MoS_2_ and WS_2_ units in the supercell used in any of the zigzag (zz-NR) and armchair (ac-NR) nanoribbons. For each of these structures, our computed interface energy is −2.27 eV, indicating that these interface formations are energetically favorable.

The structural stability of a passivated nanoribbon was examined by using the energy formula

*E*_passivation energy_ = (*E*_passivated ribbon_ − *E*_bare ribbon_ − *n*_H_ × *E*(H) − *n*_X_ × *E*(X))/2*L* (3)

where *E*_passivated ribbon_ is the total energy of the passivated ribbon, *E*_bare ribbon_ is the energy of the unpassivated (bare) ribbon, *E*(H) is taken as the half of the total energy of a H_2_ molecule, *E*(X) is half of the total energy of the X_2_ molecule, *n*_H_ is the number of H atoms, and *n*_X_ is the number of the X passivating atom. The passivation energy for the zz-NR structure in Figure 2b is −0.59 eV. This clearly suggests that this bare zz-NR in Figure 2a will become stable upon hydrogen passivation.

The high-symmetry points Γ, X and Y in Brillouin zone are marked. Due to lack of real periodicity along the *y* axis, the energy bands are flat along ΓY and we find dispersive bands along ΓX. As shown in Figure 2, we find that the zigzag (MoS_2_)_3_/(WS_2_)_3_ nanoribbon is a metal, similar to the results obtained in [32] for a MoS_2_ nanoribbon. Clearly, the H passivation of the zigzag (MoS_2_)_3_/(WS_2_)_3_ nanoribbon does not make the system semiconducting. 

#### 3.1.3. Armchair (MoS_2_)*_m_*/(WS_2_)*_n_* Lateral Nanoribbons

**Figure 3:**
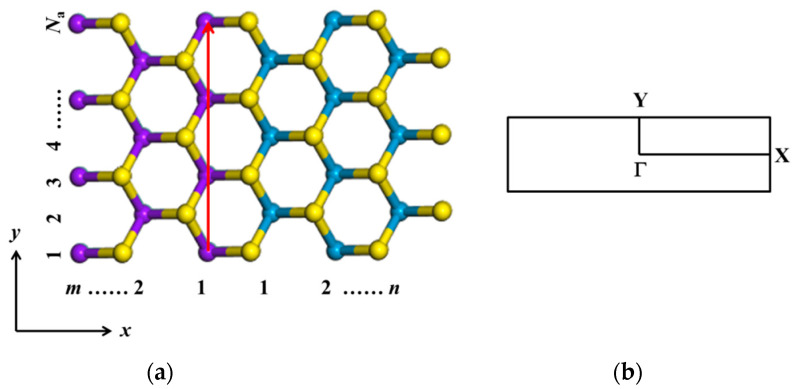
(**a**)The structure of armchair MoS_2_-WS_2_ superlattice nanoribbon. The *m* and *n* represent the components of MoS_2_ and WS_2_ in the lateral superlattice period. The *x*-axis and *y*-axis denote the period and width *N*_a_ directions, respectively. (**b**) The high-symmetry points Γ, X and Y in Brillouin zone.

Figure 3 shows the structure of armchair MoS_2_-WS_2_ lateral superlattice nanoribbon. The vertical red arrow line marks the width of armchair nanoribbon. We use the notation *N*_a_-ac-(MoS_2_)*_m_*/(WS_2_)*_n_*-NR to describe a (MoS_2_)*_m_*/(WS_2_)*_n_* armchair nanoribbon. Here the width of the armchair nanoribbon is characterized by the number of atomic layers *N*_a_ in the width direction. The numbers *m* and *n* denote the numbers of MoS_2_ and WS_2_ layers in the superlattice period. The *x*-axis is the superlattice period direction and the *y*-axis is the width direction. The high-symmetry points Γ, X and Y in the Brillouin zone are marked.

Both the symmetric and asymmetric armchair nanoribbons may lower their energy via formation of corrugation [33] or the bond reconstruction mechanism, as is widely realized for semiconductor surfaces, particularly the (001) surface of diamond structure materials and the (110) surface of zincblende materials [54]. However, in this work we have not considered any possible reconstructions of geometries discussed in the text. 

**Figure 4:**
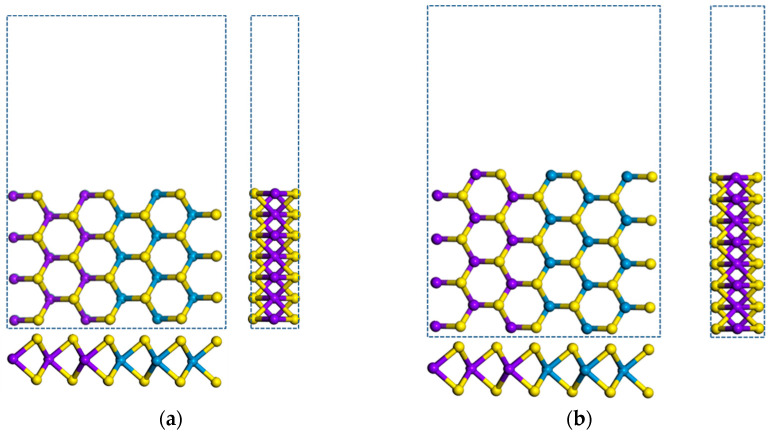

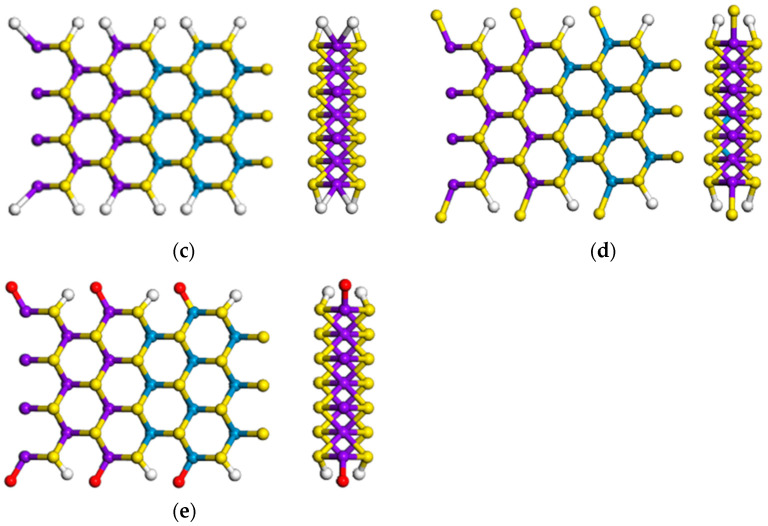
Top and side view of armchair (MoS_2_)_3_/(WS_2_)_3_ nanoribbons: (**a**) 7-ac-(MoS_2_)_3_/(WS_2_)_3_-NR-sym, (**b**) 8-ac-(MoS_2_)_3_/(WS_2_)_3_-NR-asym, (**c**) 7-ac-(MoS_2_)_3_/(WS_2_)_3_-NR-sym-H, (**d**) 7-ac-(MoS_2_)_3_/(WS_2_)_3_-NR-sym -H-S, (**e**) 7-ac-(MoS_2_)_3_/(WS_2_)_3_-NR-sym-H-O. The blue dotted rectangle shows the unit cell of armchair nanoribbon with a vacuum region along the y axis in (**a**,**b**). And (**c**–**e**) have the same vacuum region as in (**a**,**b**). Grey represents H atoms, red represents O atoms.

In order to examine the effect of edge modification on the electronic properties we first focus on the *m* = *n* = 3 superlattice structure (MoS_2_)_3_/(WS_2_)_3_. Symmetric (7-ac-(MoS_2_)_3_/(WS_2_)_3_-NR-sym) and asymmetric (8-ac-(MoS_2_)_3_/(WS_2_)_3_-NR-asym) nanoribbon structures are constructed. Bare, and H-, H-S-, H-O-passivated nanoribbons are investigated, shown in Figure 4. The 7-ac-(MoS_2_)_3_/(WS_2_)_3_-NR-sym has an odd number of atomic layers and 8-ac-(MoS_2_)_3_/(WS_2_)_3_-NR-asym has an even number of layers. For the H-passivated nanoribbon we add two H atoms to the edge Mo or W atom and one H atom to the edge S atom. For the H-S- and H-O-passivated nanoribbons, we add one S or O atom to the edge Mo or W atom and one H atom to the edge S atom.

**Figure 5:**
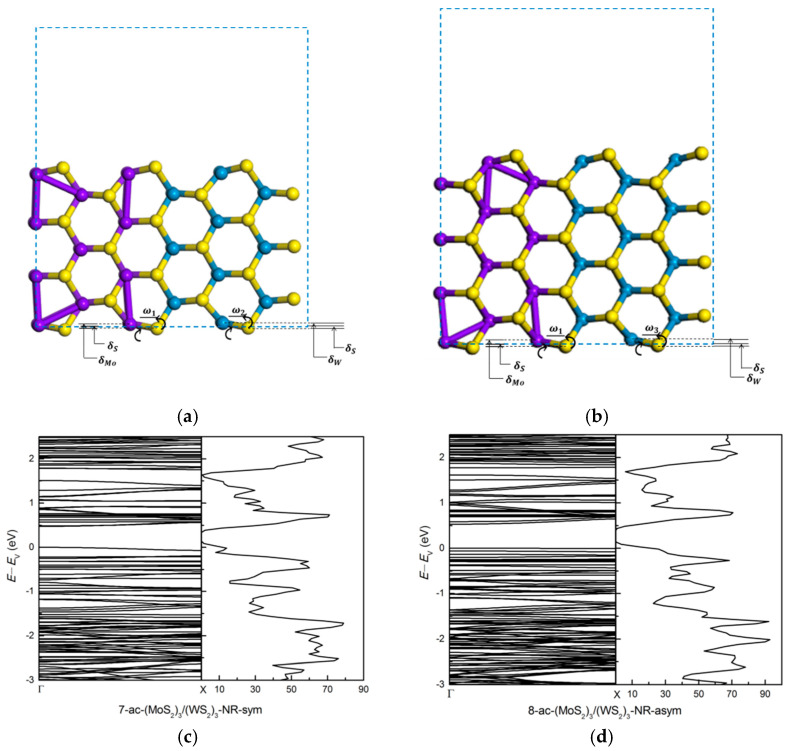
(a) The relaxed geometry of armchair (MoS_2_)_3_/(WS_2_)_3_ nanoribbons: (**a**) 7-ac-(MoS_2_)_3_/(WS_2_)_3_-NR-sym, (**b**) 8-ac-(MoS_2_)_3_/(WS_2_)_3_-NR-asym. The $\delta_{Mo}$, $\delta_{W}$, $\delta_{S}$ are the vertical displacement of the edge-layer Mo, W, S atoms which are relative to unrelaxed structures, respectively. The *ω_1_* and *ω_2_* are the angle of tilt of Mo-S bond and W-S bond from the horizontal line. The band structure and dos: (**c**) 7-ac-(MoS_2_)_3_/(WS_2_)_3_-NR-sym and (**d**) 8-ac-(MoS_2_)_3_/(WS_2_)_3_-NR-asym.

After atomic relaxation, the structure of zigzag nanoribbon does not change much. But the structure of armchair nanoribbons, both symmetric and asymmetric, has changed a lot. Compared to the S atoms, the Mo and W atoms on the edge shrink to the inside of the armchair nanoribbons, and the hexagonal ring structure is twisted and deformed, as shown in Figure 5a,b. The stability of a nanoribbon was examined by evaluating the edge energy formula in Equation (1). With *E*_edge_(asym) = +0.03 eV the asymmetric structure in Figure 4b is unstable. In contrast, with *E*_edge_(sym) = −0.26 eV the symmetric structure in Figure 4a is clearly stable. Our estimates of edge energy for these MoS_2_/WS_2_ superlattice nanoribbons are similar in trend, but different in estimate, compared to the finding in [32] for MoS_2_ armchair nanoribbons of different period and width than considered in this work.

In contrast to zigzag nanoribbons, armchair (MoS_2_)_3_/(WS_2_)_3_ nanoribbons exhibit semiconducting property. Semiconducting nature of armchair MoS_2_ nanoribbons were previously established in [32,34]. Figure 5c,d shows the bands and dos of bare sym and asym (MoS_2_)_3_/(WS_2_)_3_ armchair nanoribbon structures. The direct band gap of sym and asym structures is about 0.47 eV and 0.53 eV, respectively. These band gap values are much smaller than the band gap of the (MoS_2_)_3_/(WS_2_)_3_ superlattice presented earlier, which is caused by the formation of surface dangling bonds along the y axis. There is quite a lot of similarity and some dissimilarity between the band structures of the sym and asym nanoribbons. In particular, a few bands close to the top of the valence band (3 or 4 curves below *E*_V_) are flat for the asym structure. There are also flat bands for the sym structure, but these are further below *E*_V_. These differences are due to W and Mo being different chemical species. 

Using Equation (3) we find that H passivation stabilizes the symmetric as well the asymmetric nanoribbon structures. The stabilizing energy upon H passivation is −8.84 eV and −9.15 eV for the symmetric and asymmetric structures, respectively. That is, the asymmetric structure becomes more stable than the symmetric structure by 0.31 eV. For H-S and H-O co-passivation the relative stability energies of the asymmetric structure over the symmetric structure are, respectively, 0.31 eV and 0.36 eV. Our estimates of the results for the thin MoS_2_/WS_2_ nanoribbon are somewhat higher than those for the asymmetric MoS_2_ nanoribbon of different period and width considered in [34]. Energy gain results in passivating the armchair nanoribbon by H, H and S, and H and O are listed in Table 1.

The bands and dos of H-, H-S-, H-O-passivated 7-ac-(MoS_2_)_3_/(WS_2_)_3_-NR-sym structures are shown in Supplementary Materials Figure S1a–c. The direct band gap of the bare structure is 0.47 eV, which changes respectively to 0.63, 1.12, and 1.43 eV upon H-, H-S-, and H-O-passivation. This shows that the edge atom passivation can adjust the band gap of the armchair nanoribbon. The H-passivation opens up the band gap, but only by a small amount. The H-S and H-O passivations result in higher energy band gaps. However, the H-S-passivated structure has indirect energy band gap. The bad gap is 1.43 eV for H-O- passivation, which is close to the value for monolayer MoS_2_. This is consistent with previous reports, for example H and O edge-terminated armchair MoS_2_ exhibits a direct band gap of about 1.43 eV in [34]. We find that there is some difference in unoccupied states for the H-, H-S- and H-O-passivated 7-ac-(MoS_2_)_3_/(WS_2_)_3_-NR-sym armchair nanoribbon structures. In particular, the states around 0.75 eV for the H-passivated system have been removed for the H-S- and H-O- systems. The peak above *E*_V_ around 0.75 eV has a much bigger contribution from the *d* orbital of edge Mo and W atoms of H-passivated 7-ac-(MoS_2_)_3_/(WS_2_)_3_-NR-sym (in Supplementary Materials Figure S2), but there are no contributions from the *d* orbital of edge Mo and W atoms for the H-S- and H-O- systems in the same energy range above E_V_ (in Supplementary Materials Figures S3 and S4).

The band structure and dos for H-, H-S-, H-O-passivated 8-ac-(MoS_2_)_3_/(WS_2_)_3_-NR-asym structures are shown in Supplementary Materials Figure S5. The direct energy band gap is 0.53 eV and 0.52 eV for bare and H-passivation anr-asym, respectively, again similar to the results in [34] which quotes the band gap values of 0.61 eV and 0.60 eV for the bare and H-passivated structures, repectively. The H-S-, H-O-passivations produce larger and indirect energy band gaps. The indirect energy band gap is 1.07 eV and 1.35 eV for H-S-, H-O-passivated anr-asym, respectively. As for the 8-ac-(MoS_2_)_3_/(WS_2_)_3_-NR-asym structure, the states around 0.7 eV for the H-passivated system have been removed for the H-S- and H-O- systems. The peak above *E*_V_ around 0.7 eV has a much bigger contribution from the *d* orbital of edge Mo and W atoms of H-passivated 8-ac-(MoS_2_)_3_/(WS_2_)_3_-NR-asym (in Supplementary Materials Figure S6), but there are no contributions from the *d* orbital of edge Mo and W atoms for the H-S- and H-O- systems above *E*_V_ around 0.7 eV (in Supplementary Materials Figures S7 and S8).

### 3.2. MoS_2_/MoTe_2_ System

#### 3.2.1. (MoS_2_)_3_/(MoTe_2_)_3_ Lateral Superlattice and Alloy

The (MoS_2_)_3_/(WS_2_)_3_ lateral superlattice and nanoribbon systems considered in the previous section contain two different cations: Mo and W. Because of the similarity in lattice constant and electronic structure of MoS_2_ and WS_2_ the superlattice and nanoribbon structures constructed from these do not exhibit any extraordinary features. In contrast, superlattice and nanoribbon structures using two TMDs with different anions, such as MoS_2_ and MoTe_2_, which have different lattice constants, are expected to exhibit pronounced features in their band structure. 

**Figure 6:**
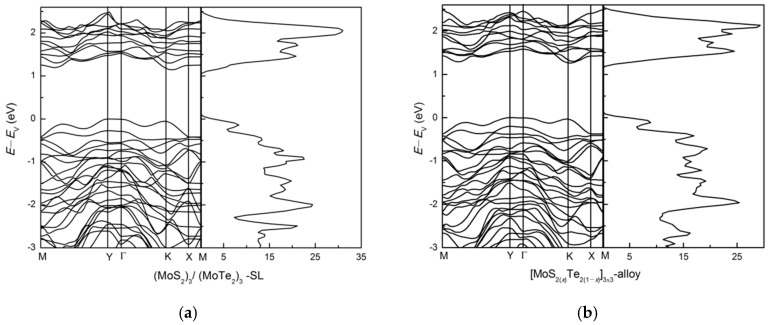
The band structure and dos: (**a**) (MoS_2_)_3_/(MoTe_2_)_3_ lateral superlattice and (**b**) [MoS_2(*x*)_Te_2(1−*x*)_]_3x3_ alloy superlattice.

To investigate such features, we first studied the band structure of the lateral superlattice (SL) as (MoS_2_)_3_/(MoTe_2_)_3_ and the alloy superlattice [MoS_2(*x*)_Te_2(1−*x*)_]_3__×3_ (*x* = 0.5) using the same unit cell. The normal SL structure has the two bond lengths arranged periodically, whereas the alloy superlattice has the two bond lengths distributed randomly throughout the structure (we chose S and Te positions in a random manner). The total energy results show that the superlattice geometry (MoS_2_)_3_/(MoTe_2_)_3_ is energetically favorable and more stable than the separated constituents alloy geometry [MoS_2(*x*)_Te_2(1−*x*)_]_3__×3_ (*x* = 0.5). The band structure and dos results are shown in Figure 6. The (MoS_2_)_3_/(MoTe_2_)_3_ superlattice is semiconductor with a band gap of 1.14 eV, close to that of monolayer MoTe_2_ (1.16 eV). The band gap of [MoS_2(*x*)_Te_2(1−*x*)_]_3__×3_ (*x* = 0.5) is 1.26 eV, larger than that of the (MoS_2_)_3_/(MoTe_2_)_3_ superlattice. After alloying, the band edge of CBM is slightly increased, so that the band gap increases about 0.12 eV. The energy band near VBM is relatively flat along ΓY and dispersive along ΓX. Due to different bond length distributions there are some differences in the band structure for the two systems. The alloy superlattice has a slightly larger band gap and its energy bands show splitting at symmetry points, notably at the zone center Γ. 

#### 3.2.2. Zigzag and Armchair (MoS_2_)_3_/(MoTe_2_)_3_ Lateral Nanoribbons

**Figure 7:**
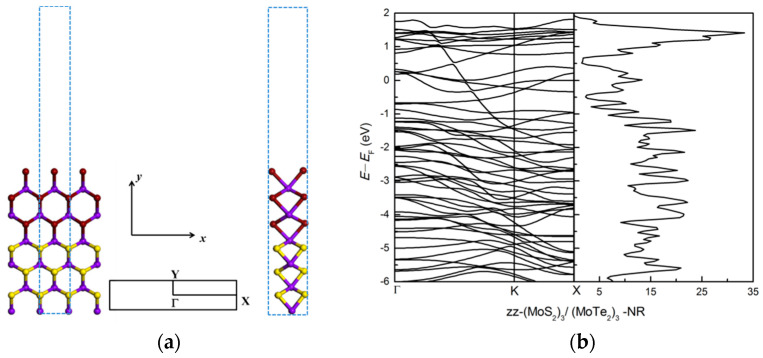
(**a**) The atomic structure of zz-(MoS_2_)_3_/(MoTe_2_)_3_-NR. The blue dotted rectangle shows the repeat unit cell in (**a**) and the high-symmetry points in Brillouin zone are marked. (**b**) The electronic band structure and density of states (dos). Purple ball represents Mo atoms, brown ball represents Te atoms, yellow ball represents S atoms.

**Figure 8:**
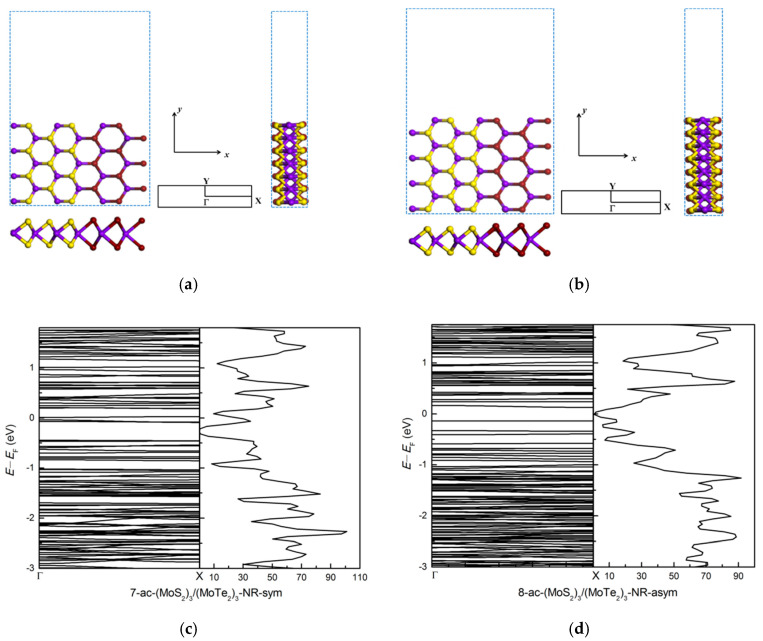
Top and side view of bare armchair (MoS_2_)_3_/(MoTe_2_)_3_ nanoribbons: (**a**) 7-ac-(MoS_2_)_3_/(MoTe_2_)_3_-NR-sym, (**b**) 8-ac-(MoS_2_)_3_/(MoTe_2_)_3_-NR-asym. The blue dotted rectangular shows the unit cell of armchair nanoribbon with a vacuum region along the *y* axis in (**a**,**b**); (**c**,**d**) show the bands and dos of bare 7-ac-(MoS_2_)_3_/(MoTe_2_)_3_-NR-sym and 8-ac-(MoS_2_)_3_/(MoTe_2_)_3_-NR-asym armchair nanoribbon structures.

Zigzag and armchair (MoS_2_)_3_/(MoTe_2_)_3_ lateral nanoribbons are investigated, with results shown in Figure 7 and Figure 8. After atomic relaxation, the structure of zigzag nanoribbon does not change much. But the structure of armchair nanoribbons has greatly changed. At the edge, compared to the anionic S atoms, the cationic Mo atoms move towards the ribbon and the anionic Te atoms move away from the ribbon, and the hexagonal ring structure is twisted and deformed. The Te atoms relax more than the S atoms.

The zigzag (MoS_2_)_3_/(MoTe_2_)_3_ nanoribbon is a metal (see Figure 7), similar to the zigzag (MoS_2_)_3_/(WS_2_)_3_ nanoribbon. However, in contrast to the zigzag (MoS_2_)_3_/(WS_2_)_3_ nanoribbon, the zigzag (MoS_2_)_3_/(MoTe_2_)_3_ nanoribbon is stable, with edge energy of −3.09 eV. The H-passivation energy is −0.34 eV.

The armchair (MoS_2_)_3_/(MoTe_2_)_3_ ribbon is stable in both symmetric and asymmetric structures, with edge energies, respectively, of −1.89 and −2.70 eV per edge atom. As shown in Table 1, the symmetric structure becomes more stable upon passivation, with energies, for H-, H-S and H-O passivations, of −8.48, −1.72 and −3.24 eV. The corresponding values for the asymmetric structure are −8.26, −1.50 and −3.10 eV. 

The interface energy between the MoS_2_ and MoTe_2_ parts, using equation (2), is −5.89 eV, −3.48 eV and −4.08 eV for the zigzag (Figure 7a), symmetric armchair (Figure 8a) and asymmetric armchair (Figure 8b) structures, respectively. It is interesting that for the MoS_2_/MoTe_2_ nanoribbon system the interface energy shows large structural dependence, while we did not find any noticeable difference for different structures of the MoS_2_/WS_2_ nanoribbons. These differences are due to different amounts of the atomic relaxation at the interface and edges for the three types of nanoribbon structures considered here (see Table 2).

**Figure 9:**
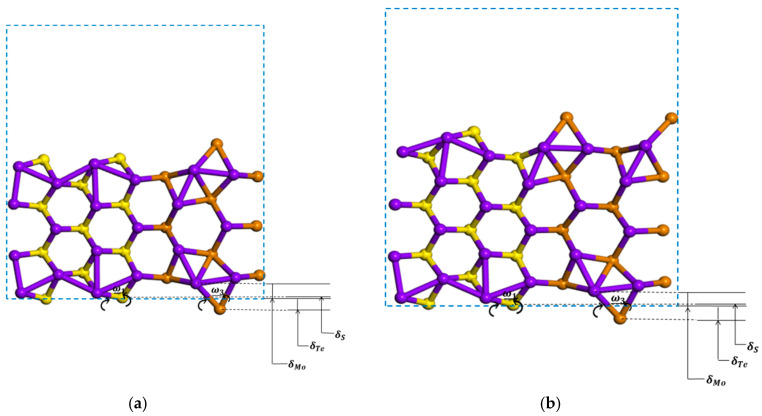
The relaxed geometries of 7-ac-(MoS_2_)_3_/(MoTe_2_)_3_-NR-sym (**a**) and 8-ac-(MoS_2_)_3_/(MoTe_2_)_3_-NR-asym (**b**). $\delta_{Mo}$, $\delta_{S}$, $\delta_{Te}$ are the vertical displacements of the edge-layer Mo, S, Te atoms relative to unrelaxed structures, respectively. *ω*_1_ and *ω*_3_ are the tilt angles for the Mo-S and Mo-Te bonds from the horizontal line.

For the armchair (MoS_2_)_3_/(MoTe_2_)_3_ ribbon structure the edge Te atoms relax much more than the S atoms for the armchair (MoS_2_)_3_/(WS_2_)_3_ ribbon structure, as shown in Figure 5 and Figure 9. Following the practice adopted for semiconductor surface relaxation [54], we show in Table 2 the vertical displacements $\delta_{Mo}$, $\delta_{W}$, $\delta_{S}$, $\delta_{Te}$ and the tilt angles *ω*_1_ (for Mo-S bond), *ω*_2_ (for W-S bond), *ω*_3_ (for Mo-Te bond) for symmetric, asymmetric (MoS_2_)_3_/(WS_2_)_3_ and (MoS_2_)_3_/(MoTe_2_)_3_ armchair nanoribbons. There is larger vertical displacement for Mo, S, Te atoms for the (MoS_2_)_3_/(MoTe_2_)_3_ armchair nanoribbon than for Mo, W, S atoms in the (MoS_2_)_3_/(WS_2_)_3_ armchair nanoribbon. Especially Te atoms and Mo atoms connected to Te have large vertical displacements. Large vertical displacements lead to large tilt angles of about 48° and 50° for the Mo-Te bond in the symmetric and asymmetric structures, respectively. Structural changes cause changes in the band structure and electronic properties. This results in large reduction in the band gap upon (MoS_2_)_3_/(MoTe_2_)_3_ ribbon formation. Figure 8 shows the bands and dos of bare symmetric (7-ac-(MoS_2_)_3_/(MoTe_2_)_3_-NR-sym) and asymmetric (8-ac-(MoS_2_)_3_/(MoTe_2_)_3_-NR-asym) armchair nanoribbon structures. Both the 7-ac-(MoS_2_)_3_/(MoTe_2_)_3_-NR-sym and 8-ac-(MoS_2_)_3_/(MoTe_2_)_3_-NR-asym systems show metallic nature. This is different from 7-ac-(MoS_2_)_3_/(WS_2_)_3_-NR-sym and 8-ac-(MoS_2_)_3_/(WS_2_)_3_-NR-asym, which are semiconductors with band gaps of 0.47 eV for sym and 0.53 eV for asym structure.

**Figure 10:**
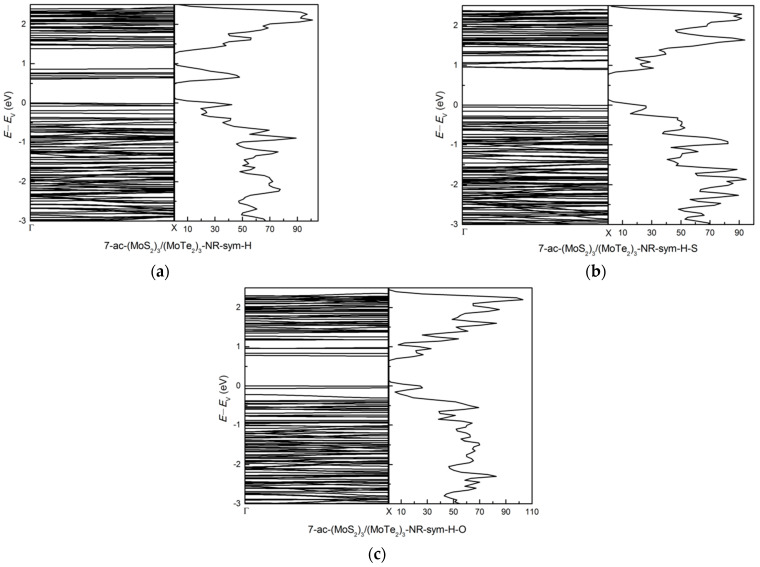
The band structure and dos: (**a**) 7-ac-(MoS_2_)_3_/(MoTe_2_)_3_-NR-sym-H, (**b**) 7-ac-(MoS_2_)_3_/(MoTe_2_)_3_-NR-sym-H-S and (**c**) 7-ac-(MoS_2_)_3_/(MoTe_2_)_3_-NR-sym-H-O.

The metallic bare symmetric armchair nanoribbon 7-ac-(MoS_2_)_3_/(MoTe_2_)_3_-NR-sym is acquires band gaps of 0.60, 0.90, and 0.76 eV for H-, H-S-, H-O-passivations, respectively, as shown in Figure 10. The H-passivated structure has direct energy band gap, but the H-S-, H-O-passivated structures have indirect energy band gap. The H-, H-S-, H-O-passivated structure opens up the band gap. The H-S-passivation produces the highest energy band gap, which is different from the (MoS_2_)_3_/(WS_2_)_3_ system. The unoccupied states of H-passivated structure are different from H-S- and H-O-passivated structures. The states around 0.75 eV for the H-passivated system decrease for H-O-passivated system and have been removed for the H-S- passivated systems. The peak above *E*_V_ around 0.75 eV has a much bigger contribution from the *d* orbital of edge Mo atoms of H-passivated 7-ac-(MoS_2_)_3_/(MoTe_2_)_3_-NR-sym, but there are no contributions from the *d* orbital of edge Mo atoms for the H-S- systems in the same energy range above *E*_V_.

**Figure 11:**
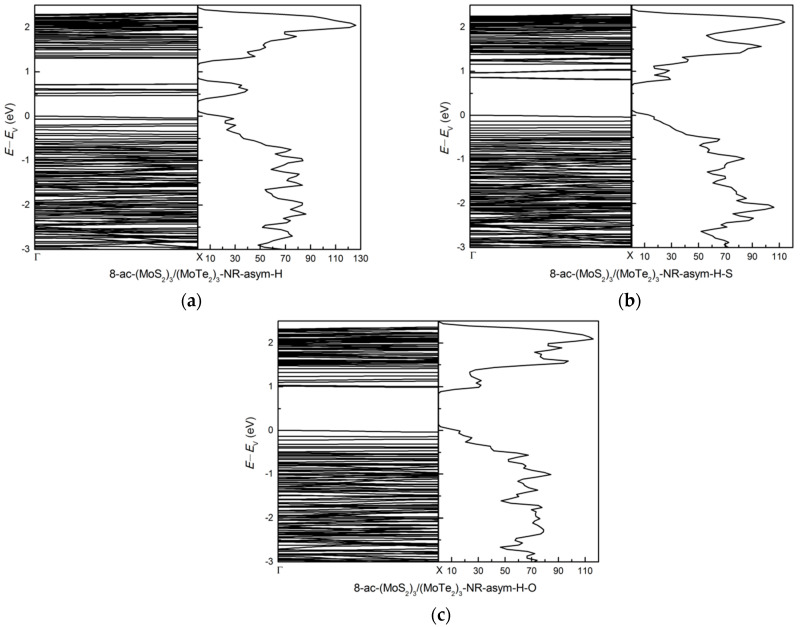
The band structure and dos: (**a**) 8-ac-(MoS_2_)_3_/(MoTe_2_)_3_-NR-asym-H, (**b**) 8-ac-(MoS_2_)_3_/(MoTe_2_)_3_-NR-asym-H-S and (**c**) 8-ac-(MoS_2_)_3_/(MoTe_2_)_3_-NR-asym-H-O.

The metallic bare asymmetric MoS_2_/MoTe_2_ armchair nanoribbon (8-ac-(MoS_2_)_3_/(MoTe_2_)_3_-NR-asym) acquires band gap values of 0.46, 0.81, and 0.98 eV for H-, H-S-, H-O-passivated asymmetric MoS_2_/MoTe_2_ armchair nanoribbon, respectively, as shown in Figure 11. The H-passivation structure has direct energy band gap. The H-S-, H-O-passivation structure has indirect energy band gap. The gap opens up for the H-, H-S-, H-O-passivated structures. The H-O-passivation produces the highest energy band gaps, similar to the (MoS_2_)_3_/(WS_2_)_3_ system. As for the 8-ac-(MoS_2_)_3_/(MoTe_2_)_3_-NR-asym structure, the states around 0.6 eV for the H-passivated system have been removed for the H-S- and H-O- systems. The peak above *E*_V_ around 0.6 eV has a much bigger contribution from the *d* orbital of edge Mo atoms of H-passivated 8-ac-(MoS_2_)_3_/(MoTe_2_)_3_-NR-asym, but there are no contributions from the *d* orbital of edge Mo atoms for the H-S- and H-O- systems above *E*_V_ around 0.6 eV. 

#### 3.2.3. Symmetric and Asymmetric MoS_2(*x*)_Te_2(1−*x*)_ Alloy (*x* = 0.5) Armchair Nanoribbons

**Figure 12:**
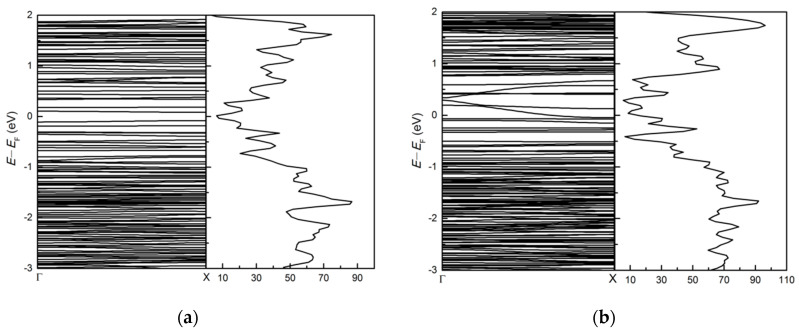
The band structure and dos of (**a**) symmetric and (**b**) asymmetric MoS_2(*x*)_Te_2(1−*x*)_ alloy (*x* = 0.5) armchair nanoribbons.

The alloy has the two bond lengths Mo-S and Mo-Te distributed randomly throughout the structure (we chose S and Te positions in a random manner). Compared to bare symmetric and asymmetric (MoS_2_)_3_/(MoTe_2_)_3_ armchair nanoribbons, the asymmetric MoS_2(*x*)_Te_2(1−*x*)_ alloy (*x* = 0.5) armchair nanoribbon and the symmetric MoS_2(*x*)_Te_2(1−*x*)_ alloy (*x* = 0.5) armchair nanoribbon are still metallic. The results are shown in Figure 12. After alloying, the band edge of CBM of symmetric MoS_2(*x*)_Te_2(1−*x*)_ alloy (*x* = 0.5) armchair nanoribbon (in Figure 12a) is slightly increased than that of symmetric (MoS_2_)_3_/(MoTe_2_)_3_ armchair nanoribbon (in Figure 8c). Due to different bond length distributions, there are some differences in the band structure for the two systems.

### 3.3. Effects of Atomic Vacancies in (MoS_2_)_3_/(WS_2_)_3_ Armchair Nanoribbon

**Figure 13:**
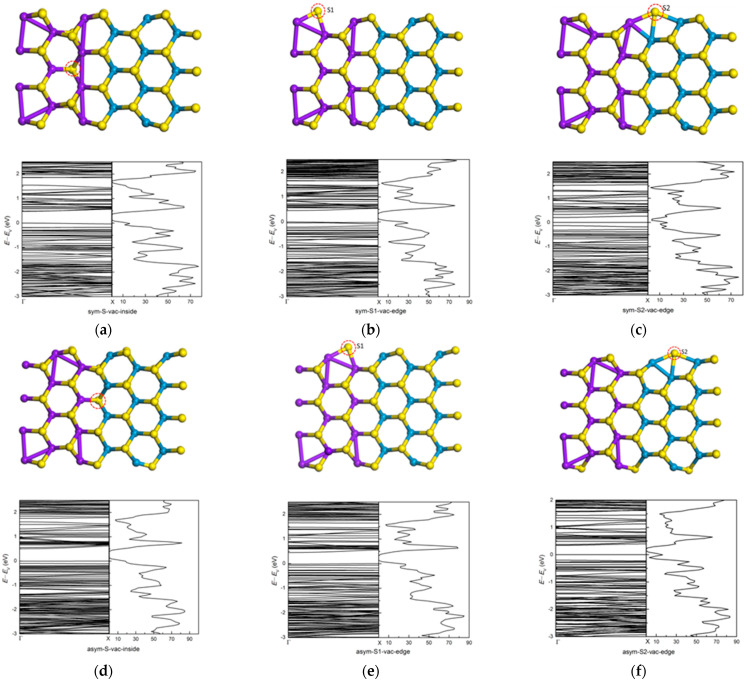
The relaxed geometry, band structure and dos of symmetric and asymmetric (MoS_2_)_3_/(WS_2_)_3_ armchair nanoribbons with a S vacancy: (**a**) symmetric structure with inside S vacancy, (**b**) symmetric structure with edge S1 vacancy and (**c**) symmetric structure with edge S2 vacancy, (**d**) asymmetric structure with inside S vacancy, (**e**) asymmetric structure with edge S1 vacancy and (**f**) asymmetric structure with edge S2 vacancy.

Point defects in a material play an important role in its physical properties. For two-dimensional materials, defects, such as impurities and vacancies, can cause local deformation and stress and have impact on their electronic, magnetic and transportation properties [27,55,56,57,58]. We consider two cases: (1) a vacancy inside the ribbon and (2) a vacancy at the ribbon edge. There is also significant difference between sym and asym structures. Figure 13 shows the bands and dos of symmetric (7-ac-(MoS_2_)_3_/(WS_2_)_3_-NR-sym) and asymmetric (8-ac-(MoS_2_)_3_/(WS_2_)_3_-NR-asym) armchair nanoribbons with an interior and an edge S vacancy. Among the edge defects, one is the defect of S connected to Mo atom (S1), and the other is the defect of S connected to W atom (S2). The edge S2 vacancy both in the symmetric and asymmetric nanoribbon produces a much-reduced band gap. The band gap is 0.48 eV and 0.46 eV for inside S vacancy and edge S1 vacancy in the symmetric (MoS_2_)_3_/(WS_2_)_3_ armchair nanoribbon, which is similar to bare symmetric (MoS_2_)_3_/(WS_2_)_3_ armchair nanoribbon (0.47 eV). We can find similar bands and dos characters around the CBM and VBM with bare symmetric (MoS_2_)_3_/(WS_2_)_3_ armchair nanoribbon. The edge S2 vacancy in the symmetric (MoS_2_)_3_/(WS_2_)_3_ armchair nanoribbon reduces the band gap from 0.47 eV to 0.20 eV. For both inside and edge S1 vacancies in the asymmetric (MoS_2_)_3_/(WS_2_)_3_ armchair nanoribbon, the band gap only changes a little, being 0.51 eV and 0.45 eV, respectively. The edge S2 vacancy in the asymmetric (MoS_2_)_3_/(WS_2_)_3_ armchair nanoribbon reduces the band gap from 0.53 eV to 0.34 eV. The bands and dos change a lot. There are new energy levels in the band gap region. The peak around *E*_V_ comes from the contribution of the *d* orbitals Mo and W atoms at the edges around the defect. 

**Figure 14:**
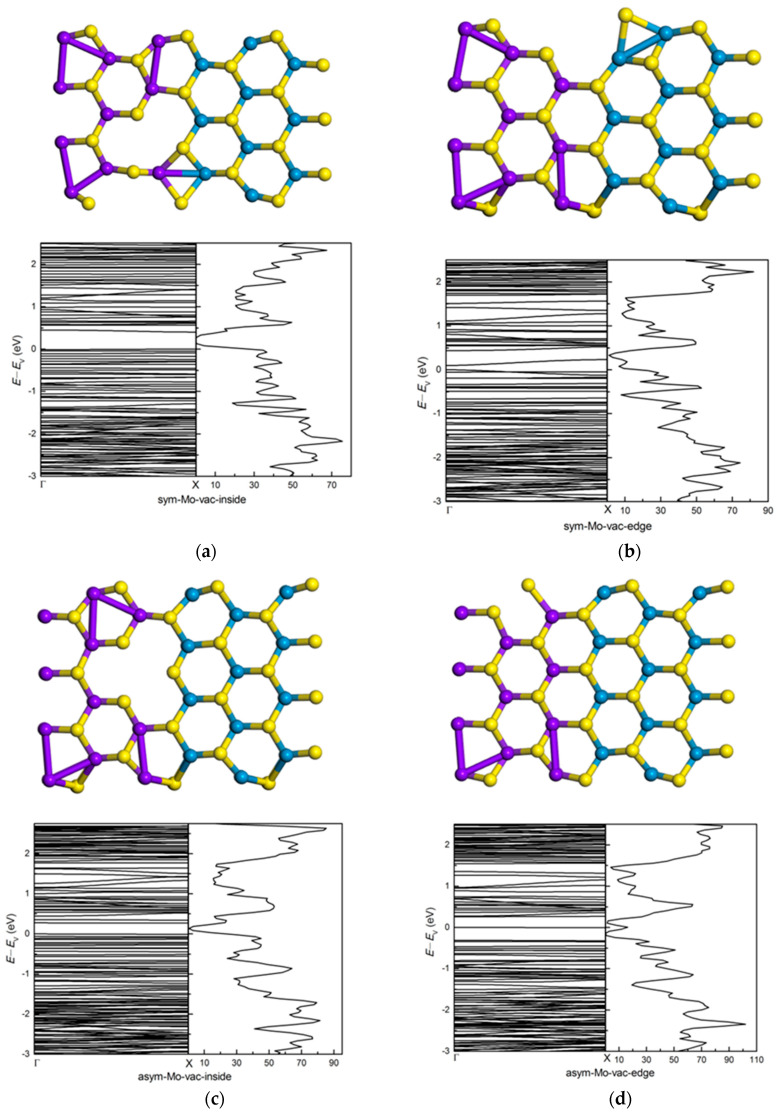
The relaxed geometry, band structure and dos of symmetric and asymmetric (MoS_2_)_3_/(WS_2_)_3_ armchair nanoribbons with Mo vacancy. (**a**) symmetric structure with inside Mo vacancy and (**b**) symmetric structure with edge Mo vacancy. (**c**) asymmetric structure with inside Mo vacancy and (**d**) asymmetric structure with edge Mo vacancy.

Figure 14 shows the bands and dos of symmetric (7-ac-(MoS_2_)_3_/(WS_2_)_3_-NR-sym) and asymmetric (8-ac-(MoS_2_)_3_/(WS_2_)_3_-NR-asym) armchair nanoribbons with inside and edge Mo vacancy. There is a clear difference in the band gap states between inside and edge Mo vacancy for symmetric structure, while there is only a little difference for asymmetric structure, inside and edge Mo vacancy both reduce the band gap. The band gap for inside Mo vacancy in symmetric (MoS_2_)_3_/(WS_2_)_3_ armchair nanoribbons is 0.40 eV. The *p* orbital of S atom and *d* orbital of Mo atoms around Mo vacancy have more contribution below the *E*_V_. The edge Mo vacancy in symmetric (MoS_2_)_3_/(WS_2_)_3_ armchair nanoribbons changes the band gap from 0.47 eV to 0.10 eV. The *p* orbital of S atom around Mo vacancy has more contribution below the *E*_V_ than that of bare symmetric (MoS_2_)_3_/(WS_2_)_3_ armchair nanoribbon. Inside and edge Mo vacancies in asymmetric (MoS_2_)_3_/(WS_2_)_3_ armchair nanoribbons both change the band gap: the value decreases to 0.27 eV and 0.26 eV, respectively. There are new energy levels around 0.28 eV and 0.35 eV for inside Mo vacancies in asymmetric (MoS_2_)_3_/(WS_2_)_3_ armchair nanoribbon. The peak comes from the contribution of the *p* orbitals of S atoms around the Mo defect. The new energy level around *E*_V_ mainly comes from the contribution of the *d* orbitals of edge W atom, and there is less contribution the *p* orbitals of the S atoms around the Mo defect.

**Figure 15:**
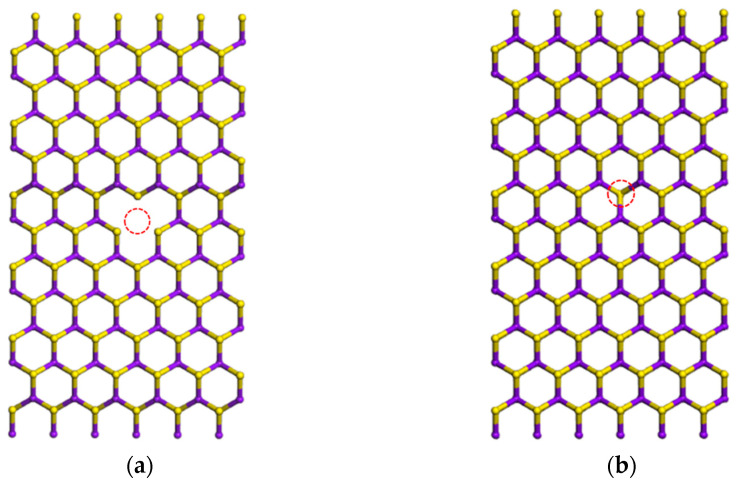
The atomic structure of MoS_2_ SL unit cell: (**a**) with a Mo vacancy in the middle, (**b**) with a S vacancy in the middle. The red circle represents the Mo vacancy and S vacancy, respectively.

There is strong vacancy-vacancy interaction in the thin-period nanoribbons considered above. In order to get a realistic understanding of the role of a single vacancy for band gap changes, we made bulk vacancy calculations using a large unit cell for MoS_2_ including 216 atoms. We created a Mo vacancy (or S vacancy) somewhere in the middle of the unit cell, as shown in Figure 15a,b. Vacancies in neighboring unit cells will be ’reasonably far’ to interact and we get vacancy related flat electronic bands inside the bulk band gap, and pdos will allow us to find out the chemical and orbital signature of that band. For Mo vacancy, impurity energy levels around 0.00 eV, 0.35 eV, 0.73 eV are found to be located near the valence band maximum (VBM) and exhibit strong local characteristics, shown in Supplementary Materials Figure S9. The energy levels at 0.00 eV and 0.35 eV are doubly degenerate. The valance band maximum (VBM) level comes from the *p* orbital of S atoms around Mo vacancy. The bottom of the conduction (CBM) level mainly comes from *d* orbitals of Mo atoms and a small part is composed of the *p* orbitals of S atoms. Thus, the impurity energy levels come from the *d* orbitals of Mo atoms and *p* orbitals of S atoms around the Mo vacancy (see Supplementary Materials Figure S10). For S vacancy, there is a doubly degenerate impurity energy level around 1.26 eV, shown in Supplementary Materials Figure S11. This impurity energy level comes from the *d* orbitals of Mo atoms around the S vacancy (see Supplementary Materials Figure S12).

### 3.4. Band Gap Variation with Ribbon Width and Period

**Figure 16:**
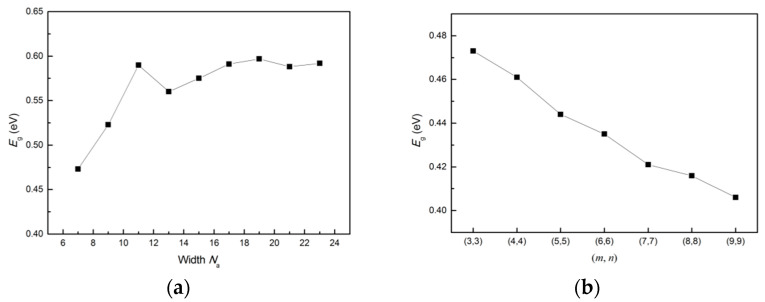
(**a**) Band gap variation in armchair nanoribbons *N*_a_-ac-(MoS_2_)_3_/(WS_2_)_3_-NR-sym (7 ≤ *N*_a_ ≤ 23) as a function of nanoribbon width *N*_a_. (**b**) Band gap variation in armchair nanoribbons 7-ac-(MoS_2_)*_m_*/(WS_2_)*_n_*-NR-sym as a function of superlattice period (*m*, *n*) in the range (3, 3) ≤ (*m*, *n*) ≤ (9, 9).

We now investigate how the band gap changes as we fix the period size and change the width for the armchair (MoS_2_)*_m_*(WS_2_)*_n_* lateral nanoribbon. We change the width *N*_a_ from 7 to 23 and obtain the relation between band gap and width as shown in Figure 16a. The band gap is oscillating as we increase the width and fix the period as (*m*, *n*) = (3, 3), and finally converges to a value about 0.59 eV, which are in agreement with the previous calculations for the armchair MoS_2_ and WS_2_ nanoribbons in [26,27,28,30,59]. Similarly, we investigate how the gap changes as we fix the width and change the period. With change in the superlattice period (*m*, *n*), the relationship between the band gap and superlattice period is shown in Figure 16b. It is not the same as increasing the width, as the band gap decreases with increasing superlattice period. This is due to reduction in the confinement effect with increase in period size from (*m*, *n*) = (3, 3) until (*m*, *n*) = (9, 9).

### 3.5. Bang Gap Variation with Biaxial Strain

**Figure 17:**
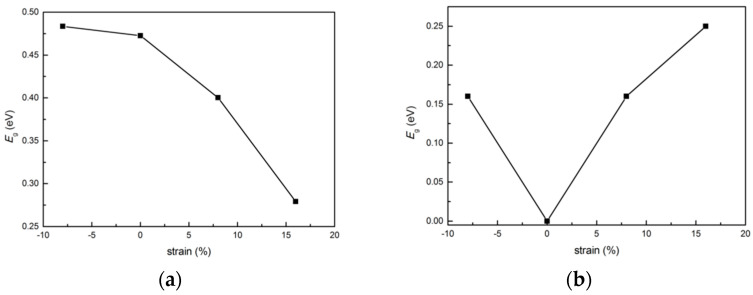
Band gap variation under strain for: (**a**) 7-ac-(MoS_2_)_3_/(WS_2_)_3_-NR-sym (left column) and (**b**) 7-ac-(MoS_2_)_3_/(MoTe_2_)_3_ -NR-sym (right column).

Application of strain can tune electronic properties of materials. We examine the effect of biaxial strain on the band gap of the nanoribbons. The electronic band structures of 7-ac-(MoS_2_)_3_/(WS_2_)_3_-NR-sym, 7-ac-(MoS_2_)_3_/(MoTe_2_)_3_-NR-sym armchair nanoribbons under tensile and compressive biaxial strain have been studied. We applied different values of biaxial strain by changing lattice constant. The band gap under strain is shown in Figure 17. The energy gap of 7-ac-(MoS_2_)_3_/(WS_2_)_3_-NR-sym increases under compressive strain and decreases under tensile strain, which is similar to the results obtained previously for monolayer MoS_2_ [42]. The energy gap of 7-ac-(MoS_2_)_3_/(MoTe_2_)_3_-NR-sym increases under both the compressive and tensile biaxial strains, which is different from the 7-ac-(MoS_2_)_3_/(MoTe_2_)_3_-NR-sym system. When the system is stretched or compressed, the relative position changes between the atoms, which affects the bonding properties and the coupling between different orbitals, so the band structure changes. The edge states play an important role in nanoribbon. The Te atom has a higher atomic number and lower electronegativity than the S atom. After atomic relaxation, the structures of 7-ac-(MoS_2_)_3_/(WS_2_)_3_-NR-sym and 7-ac-(MoS_2_)_3_/(MoTe_2_)_3_-NR-sym are very different, especially at the edge of the nanoribbon as discussed earlier, resulting in a big difference in the band structure with strain.

## 4. Conclusions

(MoS_2_)_3_/(WS_2_)_3_ and (MoS_2_)_3_/(MoTe_2_)_3_ lateral monolayer superlattices, and their zigzag and armchair nanoribbons were studied using DFT calculations. The structural, edge atom modification, vacancy effect and biaxial strain effect of (MoS_2_)_3_/(WS_2_)_3_ and (MoS_2_)_3_/(MoTe_2_)_3_ nanoribbons were investigated. The bare zig-zag and asymmetric (MoS_2_)_3_/(WS_2_)_3_ nanoribbons are unstable, but can be stabilized by H passivation. The bare symmetric (asymmetric) structure is found to be stable (unstable). Both the symmetric and asymmetric structures are stable when passivated by H atoms, or co-passivated by H and S, or by H and O atoms; however, the asymmetric structure is relatively more stable. In contrast to the (MoS_2_)_3_/(WS_2_)_3_ nanoribbons, zig-gag and armchair (MoS_2_)_3_/(MoTe_2_)_3_ nanoribbons are stable with or without passivation. Because of large lattice mismatch the band structure of (MoS_2_)_3_/(MoTe_2_)_3_ superlattice is quite different from that of (MoS_2_)_3_/(WS_2_)_3_. The band gaps open up when the (MoS_2_)_3_/(WS_2_)_3_ and (MoS_2_)_3_/(MoTe_2_)_3_ armchair nanoribbons are passivated by H, S and O atoms. In particular, (MoS_2_)_3_/(WS_2_)_3_ ANR-H-O exhibits a remarkably large band gap change. The band gap of the (MoS_2_)_3_/(WS_2_)_3_ nanoribbon oscillates as we increase the width and fix the period as (*m*, *n*) = (3, 3), and finally converges to a value about 0.59 eV. When we fix the width and change the period, the band gap decreases with increasing superlattice period. The band gap with the edge S vacancy connecting to the W atom is much smaller than the S vacancy connecting to the Mo atom. In contrast, inside and edge Mo vacancies both reduce the band gap. The electronic orbitals of atoms around the Mo vacancy play an important role in characterizing the VBM and CBM and impurity levels. Under tensile and compressive biaxial strain, the structures of 7-ac-(MoS_2_)_3_/(WS_2_)_3_-NR-sym and 7-ac-(MoS_2_)_3_/(MoTe_2_)_3_-NR-sym are very different, resulting in a big difference in the band structure with strain. 

## Figures and Tables

**Table 1:** Edge, interface and passivation energies per interface unit cell of zig-zag and armichair (MoS_2_)_3_/(MoW_2_)_3_ and (MoS_2_)_3_/(MoTe_2_)_3_ superlattice nanoribbons. See Equations (1)–(3) for the energy definitions.

System	Edge Energy (eV)	Interface Energy (eV)	Passivation Energy (eV)
zz-(MoS_2_)_3_/(WS_2_)_3_-NR	+0.89	−2.27	
zz-(MoS_2_)_3_/(WS_2_)_3_-NR-H			−0.59
7-ac-(MoS_2_)_3_/(WS_2_)_3_-NR-sym	−0.26	−2.28	
7-ac-(MoS_2_)_3_/(WS_2_)_3_-NR-sym-H			−8.84
7-ac-(MoS_2_)_3_/(WS_2_)_3_-NR-sym-H-S			−2.13
7-ac-(MoS_2_)_3_/(WS_2_)_3_-NR-sym-H-O			−3.86
8-ac-(MoS_2_)_3_/(WS_2_)_3_-NR-asym	+3.10	−2.28	
8-ac-(MoS_2_)_3_/(WS_2_)_3_-NR-asym-H			−9.15
8-ac-(MoS_2_)_3_/(WS_2_)_3_-NR-asym-H-S			−2.44
8-ac-(MoS_2_)_3_/(WS_2_)_3_-NR-asym-H-O			−4.22
zz-(MoS_2_)_3_/(MoTe_2_)_3_-NR	−3.09	−5.89	
zz-(MoS_2_)_3_/(MoTe_2_)_3_-NR-H			−0.34
7-ac-(MoS_2_)_3_/(MoTe _2_)_3_-NR-sym	−1.89	−3.48	
7-ac-(MoS_2_)_3_/(MoTe _2_)_3_-NR-sym-H			−8.48
7-ac-(MoS_2_)_3_/(MoTe _2_)_3_-NR-sym-H-S			−1.72
7-ac-(MoS_2_)_3_/(MoTe _2_)_3_-NR-sym-H-O			−3.24
8-ac-(MoS_2_)_3_/(MoTe_2_)_3_-NR-asym	−2.70	−4.08	
8-ac-(MoS_2_)_3_/(MoTe_2_)_3_-NR-asym-H			−8.26
8-ac-(MoS_2_)_3_/(MoTe_2_)_3_-NR-asym-H-S			−1.50
8-ac-(MoS_2_)_3_/(MoTe_2_)_3_-NR-asym-H-O			−3.10

**Table 2:** The vertical displacements $\delta_{W},\;\delta_{S}$, $\delta_{Te}$ and the tilt angles *ω*_1_ (for Mo-S bond), *ω*_2_ (for W-S bond), *ω*_3_ (for Mo-Te bond) for symmetric, asymmetric (MoS_2_)_3_/(WS_2_)_3_ and (MoS_2_)_3_/(MoTe_2_)_3_ armchair nanoribbons. $\delta_{Mo-S}$, $\delta_{W-S}$ and $\delta_{Mo-Te}$ are defined as |$\delta_{Mo}$-$\delta_{S}$|, |$\delta_{W}$-$\delta_{S}$| and |$\delta_{Mo}$-$\delta_{Te}$|, respectively (see Figure 5 and Figure 9).

Systems		$\delta_{Mo}$ (Å)	$\delta_{W}$ (Å)	$\delta_{S}$ (Å)	$\delta_{Te}$ (Å)	$\delta_{Mo-s}$ (Å)	$\delta_{W-s}$ (Å)	$\delta_{Mo-Te}$ (Å)	*ω*_1_	*ω*_2_	*ω*_3_
**(MoS_2_)_3_/(WS_2_)_3_** **_ANR**	sym	0.266	0.271	−0.074 (S-Mo) −0.086 (S-W)		0.340	0.357		10°	12°	
	asym	0.275	0.277	−0.077 (S-Mo) −0.088 (S-W)		0.352	0.365		11°	13°	
**(MoS_2_)_3_/(MoTe_2_)_3_** **_ANR**	sym	0.469 (Mo-S) 1.020 (Mo-Te)		0.114	−0.803	0.355		1.823	11°		48°
	asym	0.509 (Mo-S) 0.969 (Mo-Te)		0.057	−0.869	0.452		1.838	16°		50°

## Data Availability

No data sets were generated or analyzed during the current study.

## Supplementary Materials

The supplementary material contains the band structure, dos and pdos of H-, H-S- and H-O-passivated symmetric and asymmetric (MoS_2_)_3_/(MoTe_2_)_3_ armchair nanoribbons. The band structure, dos and pdos of MoS_2_ SL with a Mo or S vacancy in the middle. The following are available online at https://www.mdpi.com/2079-4991/11/2/534/s1, Figure S1: The band structure and dos: (a) 7-ac-(MoS_2_)_3_/(WS_2_)_3_-NR-sym-H, (b) 7-ac-(MoS_2_)_3_/(WS_2_)_3_-NR-sym-H-S and (c) 7-ac-(MoS_2_)_3_/(WS_2_)_3_-NR-sym-H-O, Figure S2: (a) The relaxed geometry of 7-ac-(MoS_2_)_3_/(WS_2_)_3_-NR-sym-H, edge and inside atoms for Mo and W are labeled. The partial density of states (pdos) for (b) inside Mo6 atom; (c) edge Mo10 atom; (d) edge Mo11 atom; (e) inside W15 atom; (f) edge W21 atom of 7-ac-(MoS_2_)_3_/(WS_2_)_3_-NR-sym-H nanoribbon, Figure S3: (a) The relaxed geometry of 7-ac-(MoS_2_)_3_/(WS_2_)_3_-NR-sym-H-S, edge and inside atoms for Mo and W are labeled. The partial density of states (pdos) for (b) inside Mo6 atom; (c) edge Mo10 atom; (d) edge Mo11 atom; (e) inside W15 atom; (f) edge W21 atom of 7-ac-(MoS_2_)_3_/(WS_2_)_3_-NR-sym-H-S nanoribbon, Figure S4: (a) The relaxed geometry of 7-ac-(MoS_2_)_3_/(WS_2_)_3_-NR-sym-H-O, edge and inside atoms for Mo and W are labeled. The partial density of states (pdos) for (b) inside Mo6 atom; (c) edge Mo10 atom; (d) edge Mo11 atom; (e) inside W15 atom; (f) edge W21 atom of 7-ac-(MoS_2_)_3_/(WS_2_)_3_-NR-sym-H-O nanoribbon, Figure S5: The band structure and dos: (a) 8-ac-(MoS_2_)_3_/(WS_2_)_3_-NR-asym-H, (b) 8-ac-(MoS_2_)_3_/(WS_2_)_3_-NR-asym-H-S and (c) 8-ac-(MoS_2_)_3_/(WS_2_)_3_-NR-asym-H-O, Figure S6: (a) The relaxed geometry of 8-ac-(MoS_2_)_3_/(WS_2_)_3_-NR-asym-H, edge and inside atoms for Mo and W are labeled. The partial density of states (pdos) for (b) inside Mo9 atom; (c) edge Mo11 atom; (d) inside W16 atom; (e) edge W22 atom of 8-ac-(MoS_2_)_3_/(WS_2_)_3_-NR-asym-H nanoribbon, Figure S7: (a) The relaxed geometry of 8-ac-(MoS_2_)_3_/(WS_2_)_3_-NR-asym-H-S, edge and inside atoms for Mo and W are labeled. The partial density of states (pdos) for (b) inside Mo9 atom; (c) edge Mo11 atom; (d) inside W16 atom; (e) edge W22 atom of 8-ac-(MoS_2_)_3_/(WS_2_)_3_-NR-asym-H-S nanoribbon, Figure S8: (a) The relaxed geometry of 8-ac-(MoS_2_)_3_/(WS_2_)_3_-NR-asym-H-O, edge and inside atoms for Mo and W are labeled. The partial density of states (pdos) for (b) inside Mo9 atom; (c) edge Mo11 atom; (d) inside W16 atom; (e) edge W22 atom of 8-ac-(MoS_2_)_3_/(WS_2_)_3_-NR-asym-H-O nanoribbon, Figure S9: The band structure and dos of MoS_2_ SL with a Mo vacancy in the middle, Figure S10: (a) The geometry of MoS_2_ SL with a Mo vacancy in the middle and the atoms around Mo vacancy are labeled. The partial density of states (pdos) for atoms around Mo vacancy: (b) Mo25 atom; (c) Mo26 atom; (d) Mo28 atom; (e) Mo30 atom; (f) Mo44 atom; (g) Mo47 atom; (h) S106 atom; (i) S118 atom; (j) S180 atom of MoS_2_ SL with a Mo vacancy in the middle, Figure S11: The band structure and dos of MoS_2_ SL with a S vacancy in the middle, Figure S12: (a) The geometry of MoS_2_ SL with a S vacancy in the middle and the atoms around S vacancy are labeled. The partial density of states (pdos) for atoms around S vacancy: (b) Mo25 atom; (c) Mo27 atom; (d) Mo28 atom; (e) S180 atom of MoS_2_ SL with a S vacancy in the middle.
