# Supplementary material for: Tunable Electronic Properties of Lateral Monolayer Transition Metal Dichalcogenide Superlattice Nanoribbons
Source: Nanomaterials (Basel). 2021 Feb 19;11(2):534. doi: 10.3390/nano11020534 (PMC7923096; doi:10.3390/nano11020534)
Supplement: Supplementary file 1 [file nanomaterials-11-00534-s001.zip › nanomaterials-1084526-supplementary.pdf]

nanomaterials

Supplementary Materials

Tunable Electronic Properties of Lateral Monolayer Transition Metal Dichalcogenide Superlattice Nanoribbons

Jinhua Wang [1,2,]* and G. P. Srivastava [2]

[1] School of Science, Tianjin University of Technology and Education, Tianjin, 300222, China
[2] School of Physics, University of Exeter, Exeter EX4 4QL, UK; g.p.srivastava@exeter.ac.uk
* Correspondence: jinhuawang@tute.edu.cn

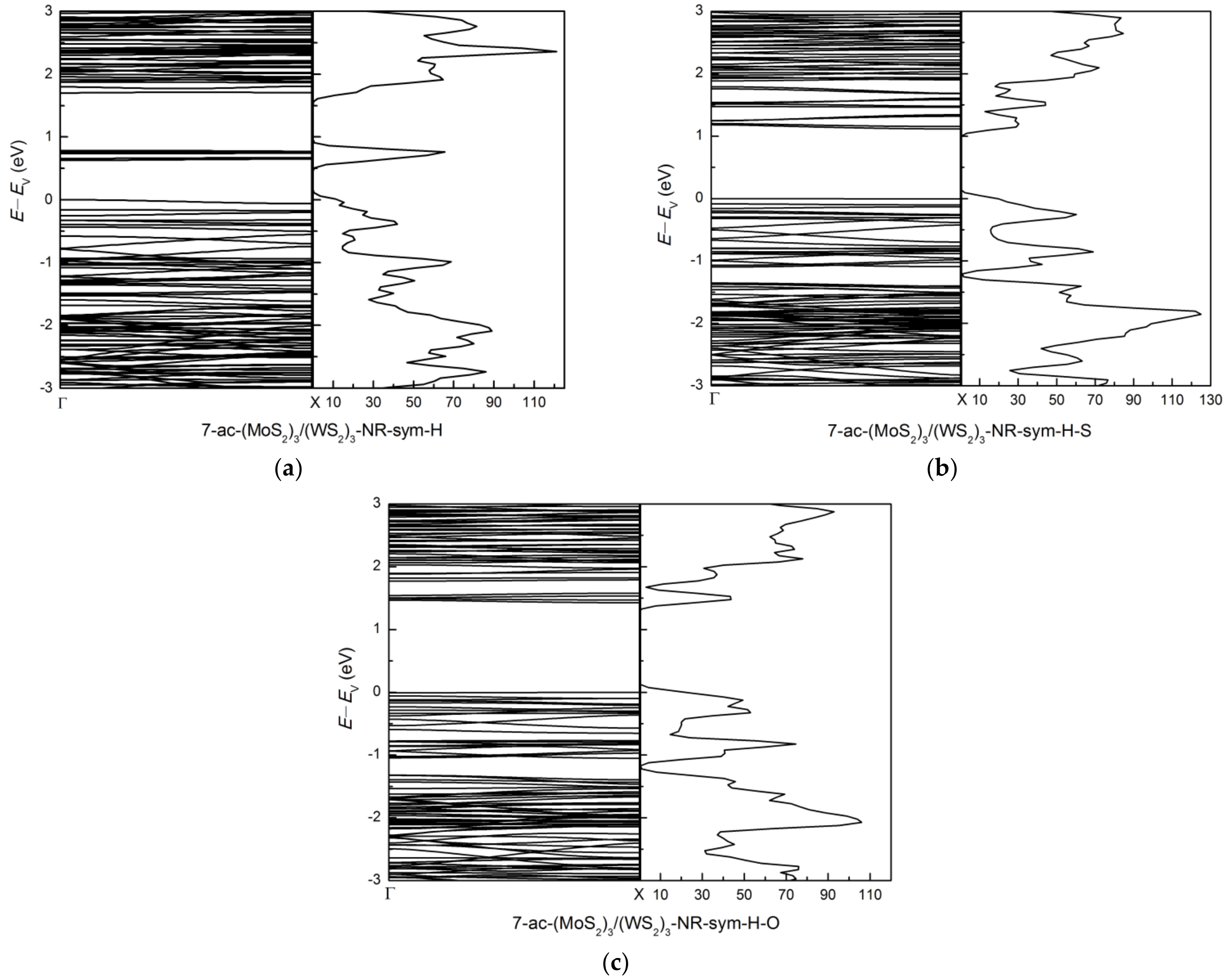

Figure S1. The band structure and dos: (**a**) 7-ac-$(MoS_2)_3/(WS_2)_3$-NR-sym-H, (**b**) 7-ac-$(MoS_2)_3/(WS_2)_3$-NR-sym-H-S and (**c**) 7-ac-$(MoS_2)_3/(WS_2)_3$-NR-sym-H-O.

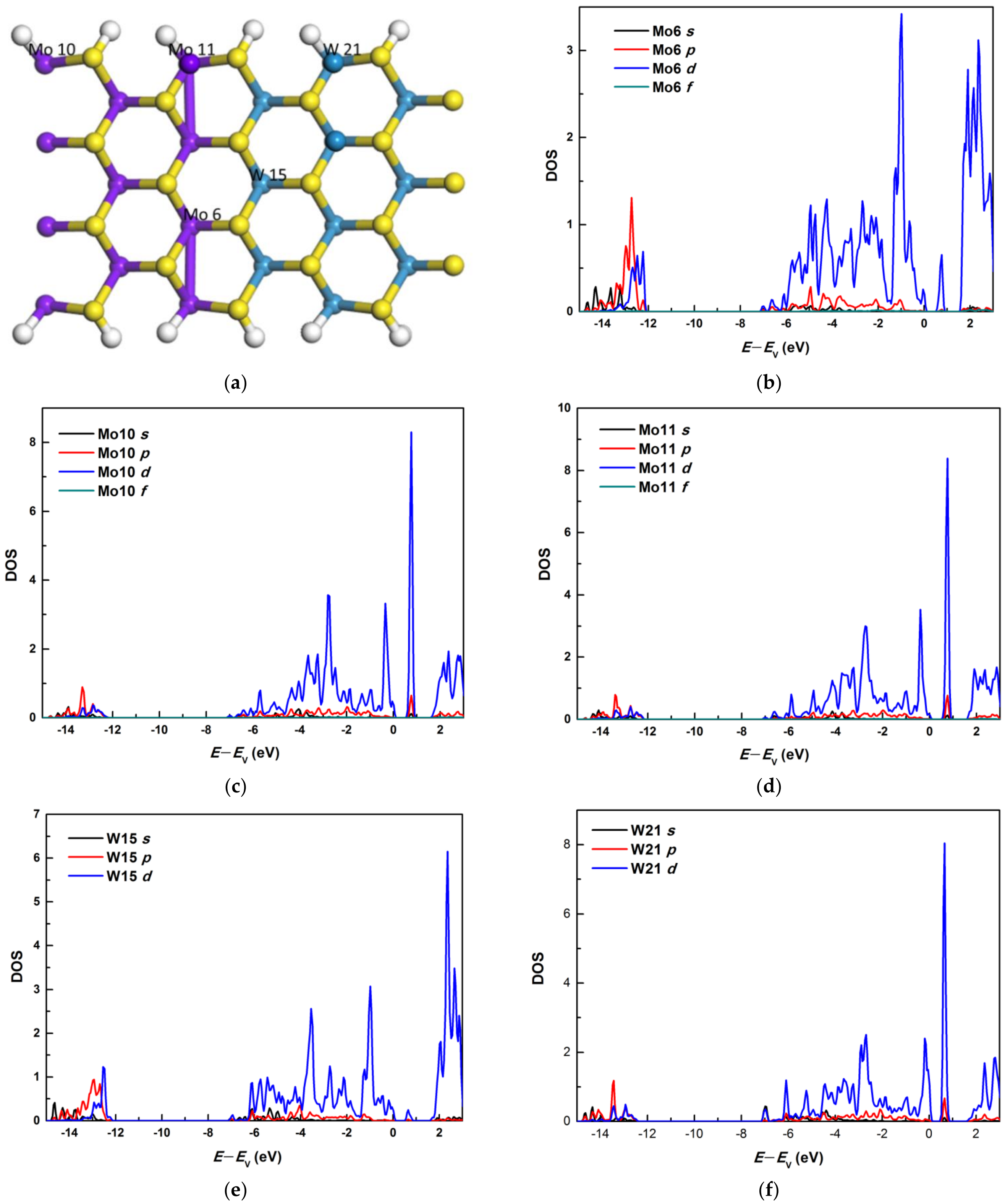

Figure S2. (**a**)The relaxed geometry of 7-ac-$(MoS_2)_3/(WS_2)_3$-NR-sym-H, edge and inside atoms for Mo and W are labeled. The partial density of states (pdos) for (**b**) inside Mo6 atom; (**c**) edge Mo10 atom; (**d**) edge Mo11 atom; (**e**) inside W15 atom; (**f**) edge W21 atom of 7-ac-$(MoS_2)_3/(WS_2)_3$-NR-sym-H nanoribbon.

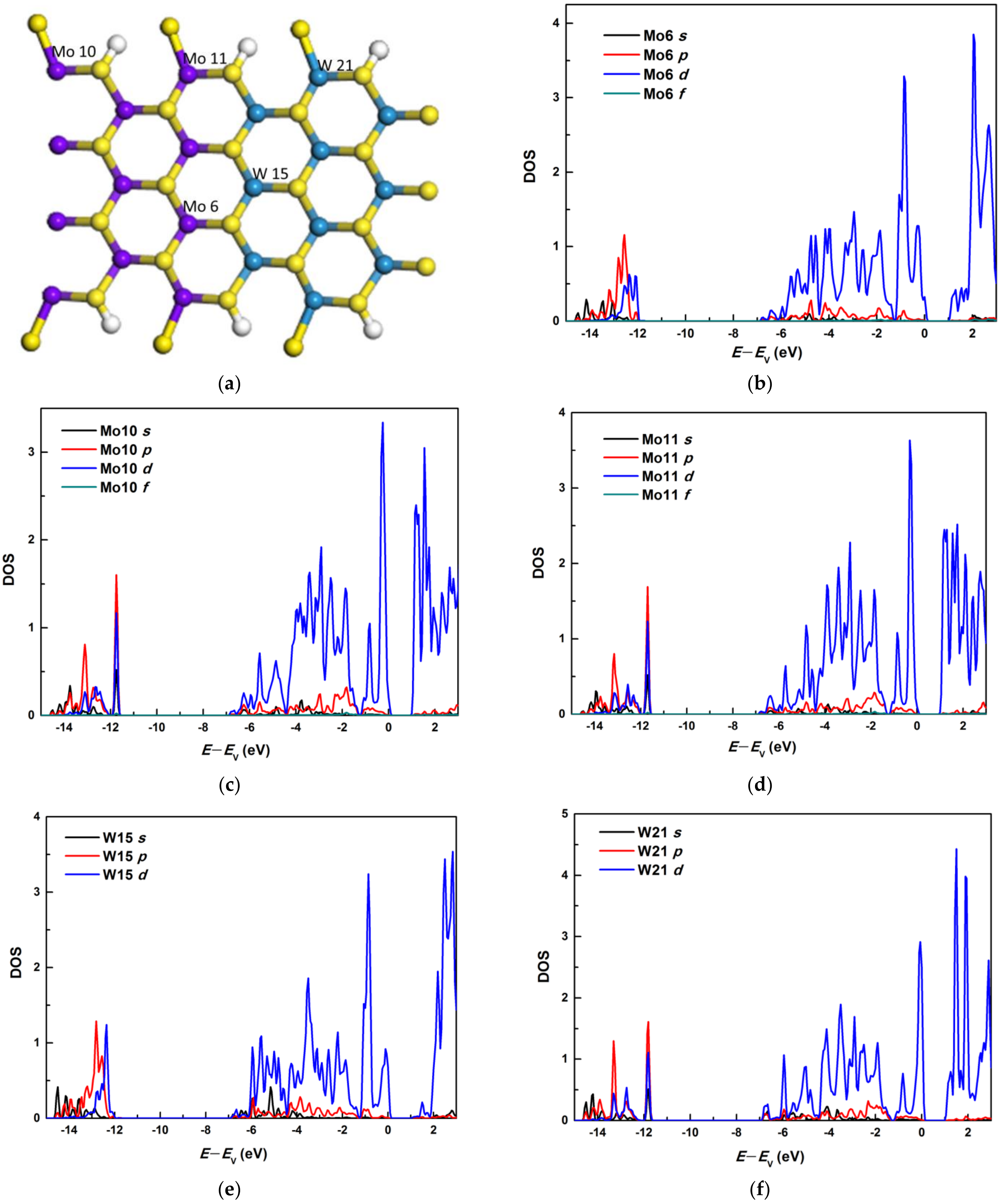

Figure S3. (**a**)The relaxed geometry of 7-ac-$(MoS_2)_3/(WS_2)_3$-NR-sym-H-S, edge and inside atoms for Mo and W are labeled. The partial density of states (pdos) for (**b**) inside Mo6 atom; (**c**) edge Mo10 atom; (**d**) edge Mo11 atom; (**e**) inside W15 atom; (**f**) edge W21 atom of 7-ac-$(MoS_2)_3/(WS_2)_3$-NR-sym-H-S nanoribbon.

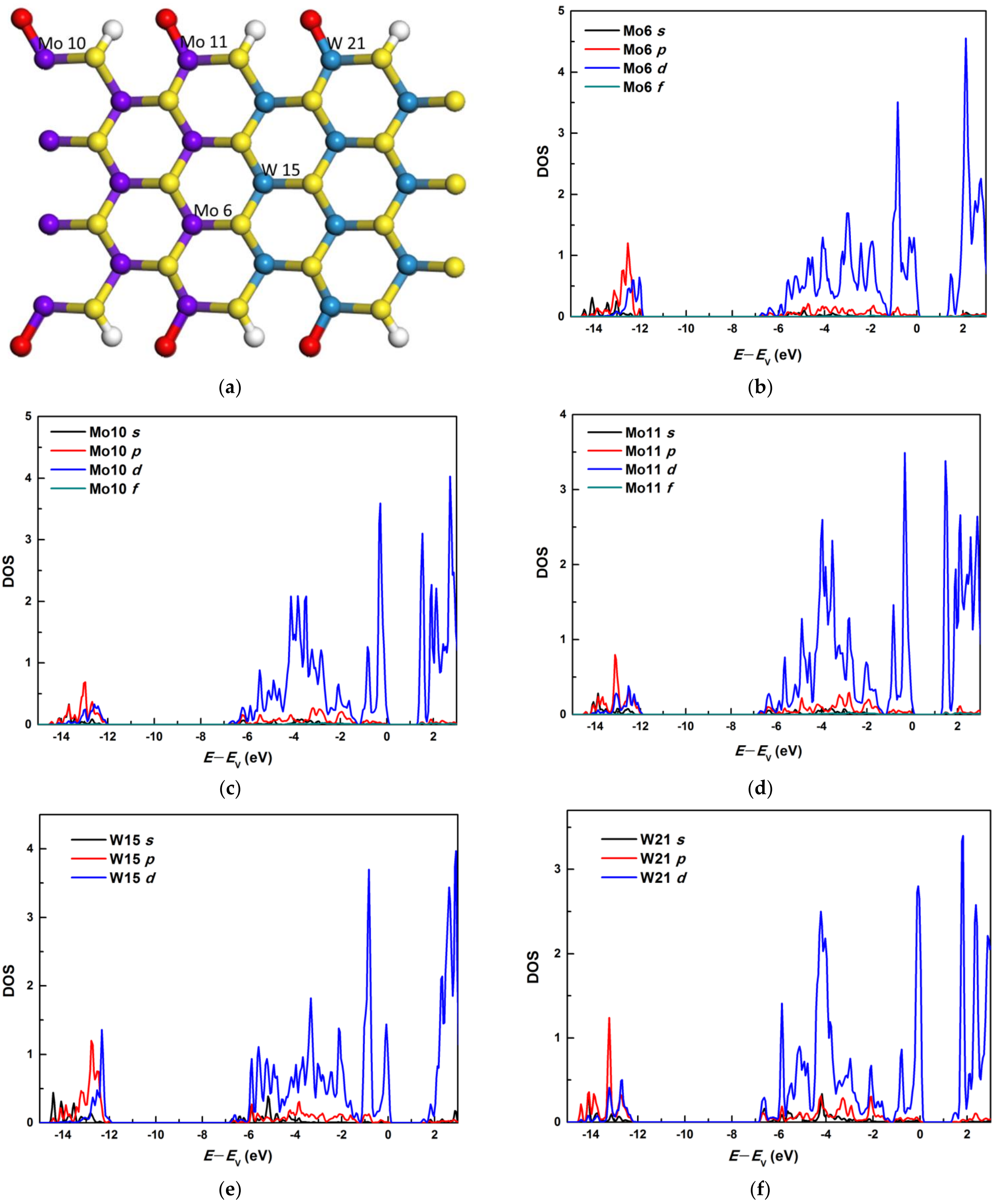

Figure S4. (**a**)The relaxed geometry of 7-ac-$(MoS_2)_3/(WS_2)_3$-NR-sym-H-O, edge and inside atoms for Mo and W are labeled. The partial density of states (pdos) for (**b**) inside Mo6 atom; (**c**) edge Mo10 atom; (**d**) edge Mo11 atom; (**e**) inside W15 atom; (**f**) edge W21 atom of 7-ac-$(MoS_2)_3/(WS_2)_3$-NR-sym-H-O nanoribbon.

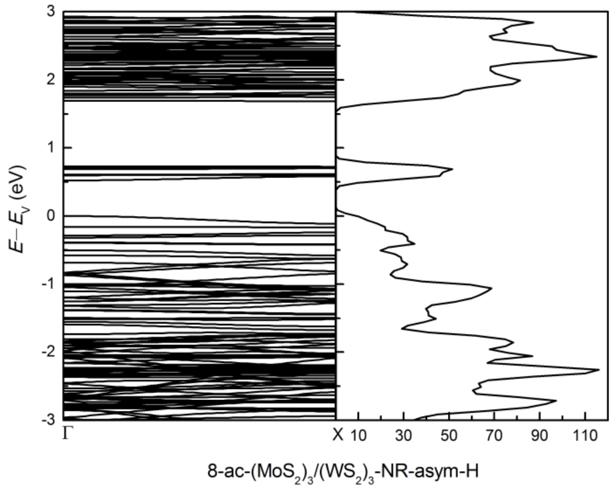

(**a**)

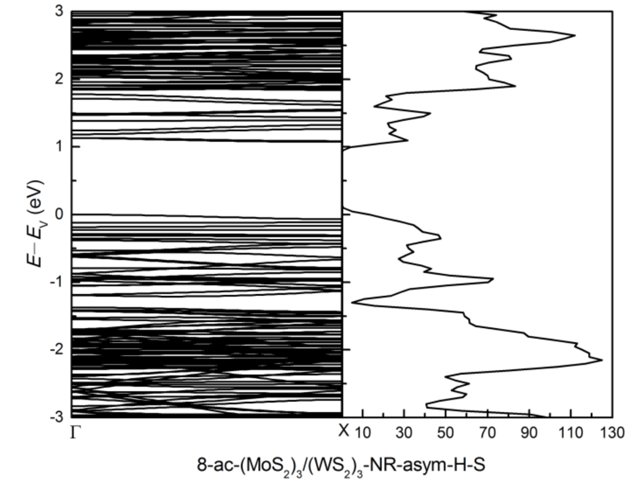

(**b**)

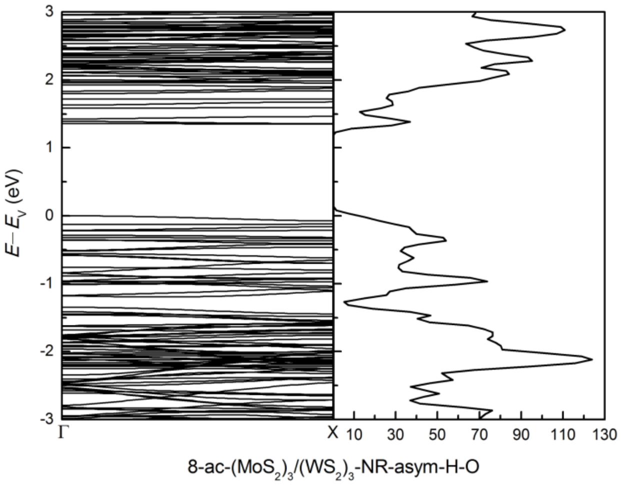

(**c**)

Figure S5. The band structure and dos: (**a**) 8-ac-$(MoS_2)_3/(WS_2)_3$-NR-asym-H, (**b**) 8-ac-$(MoS_2)_3/(WS_2)_3$-NR-asym-H-S and (**c**) 8-ac-$(MoS_2)_3/(WS_2)_3$-NR-asym-H-O.

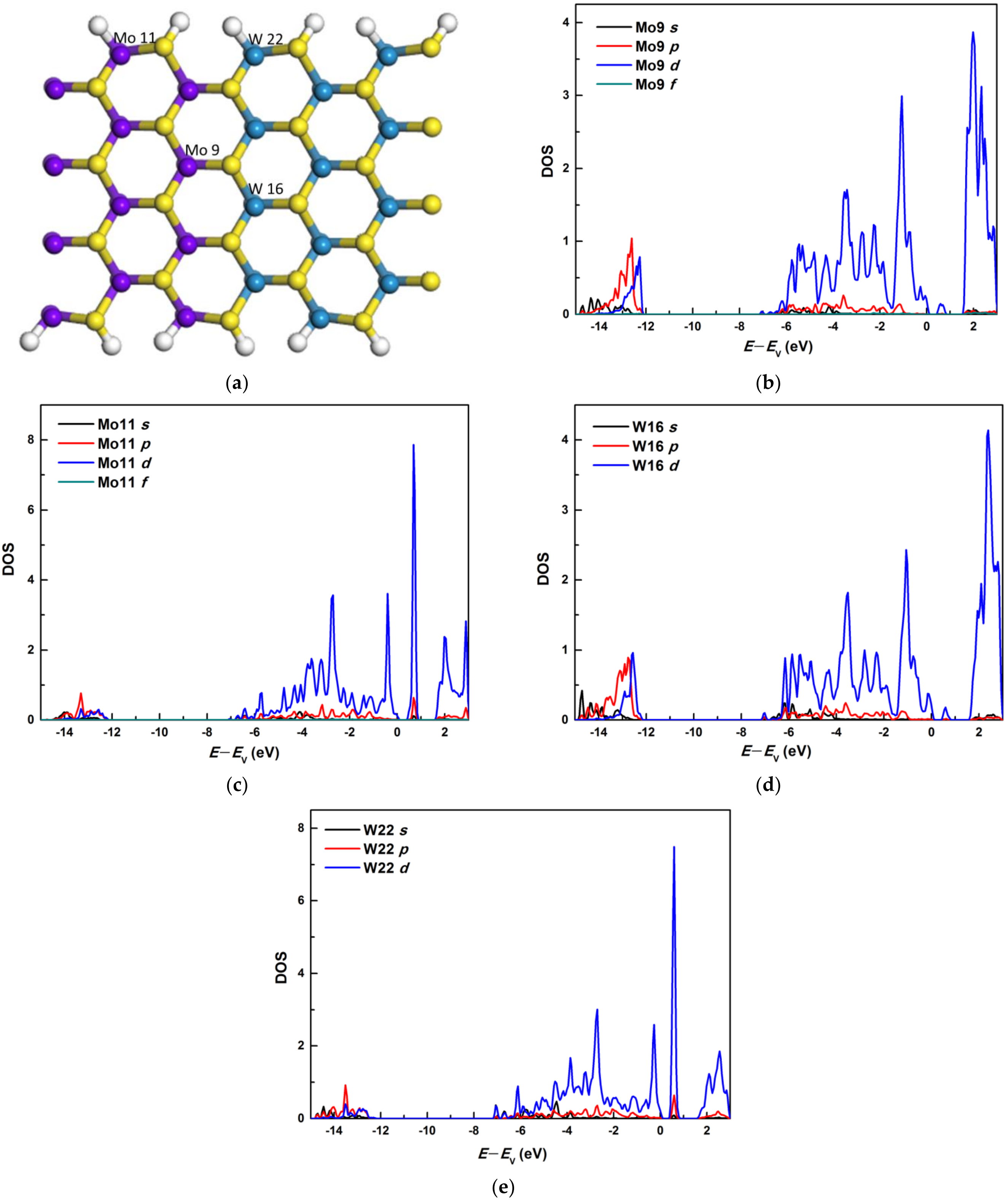

Figure S6. (**a**)The relaxed geometry of 8-ac-$(MoS_2)_3/(WS_2)_3$-NR-asym-H, edge and inside atoms for Mo and W are labeled. The partial density of states (pdos) for (**b**) inside Mo9 atom; (**c**) edge Mo11 atom; (**d**) inside W16 atom; (**e**) edge W22 atom of 8-ac-$(MoS_2)_3/(WS_2)_3$-NR-asym-H nanoribbon.

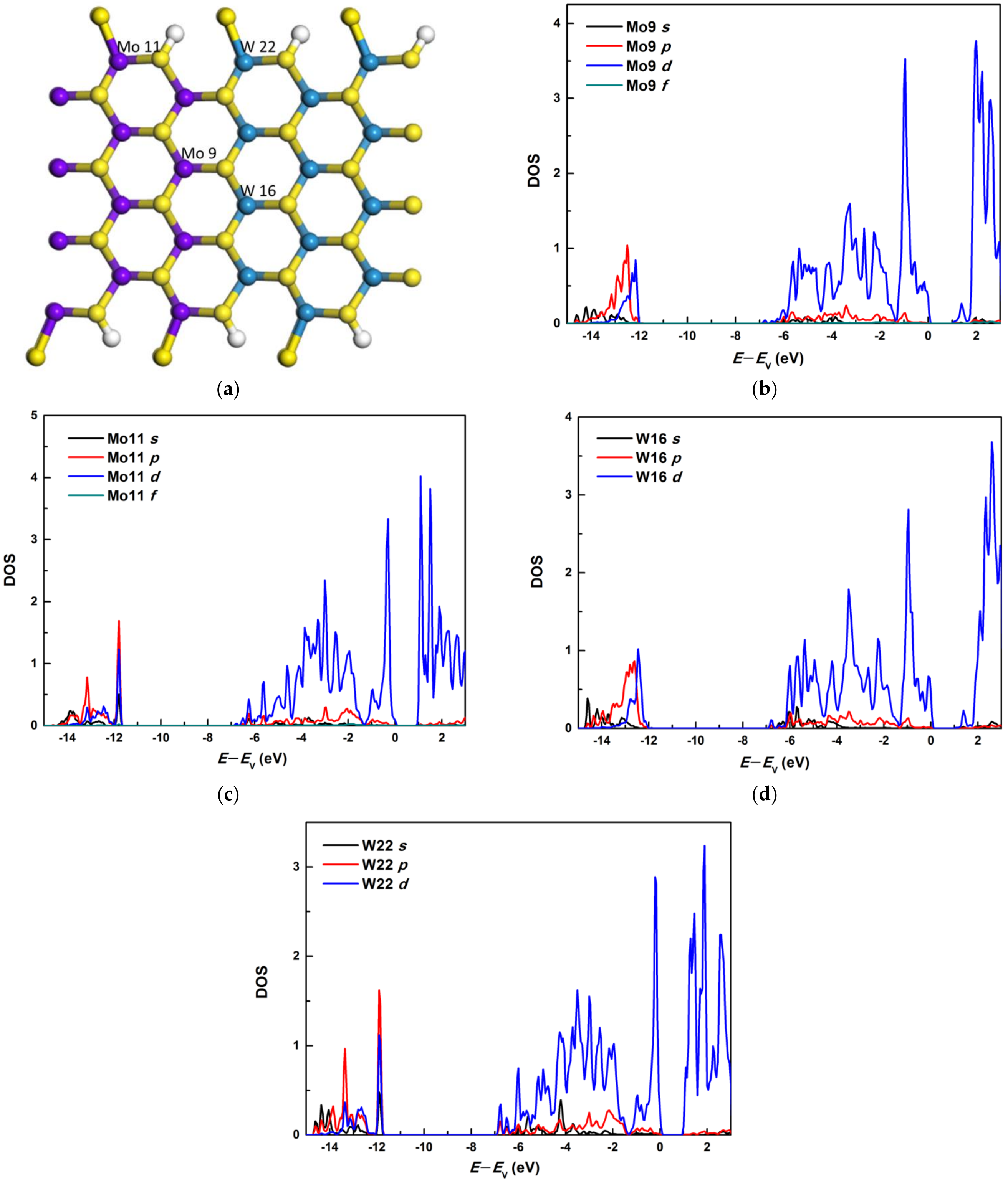

(e)

Figure S7. (**a**)The relaxed geometry of 8-ac-$(MoS_2)_3/(WS_2)_3$-NR-asym-H-S, edge and inside atoms for Mo and W are labeled. The partial density of states (pdos) for (**b**) inside Mo9 atom; (**c**) edge Mo11 atom; (**d**) inside W16 atom; (**e**) edge W22 atom of 8-ac-$(MoS_2)_3/(WS_2)_3$-NR-asym-H-S nanoribbon.

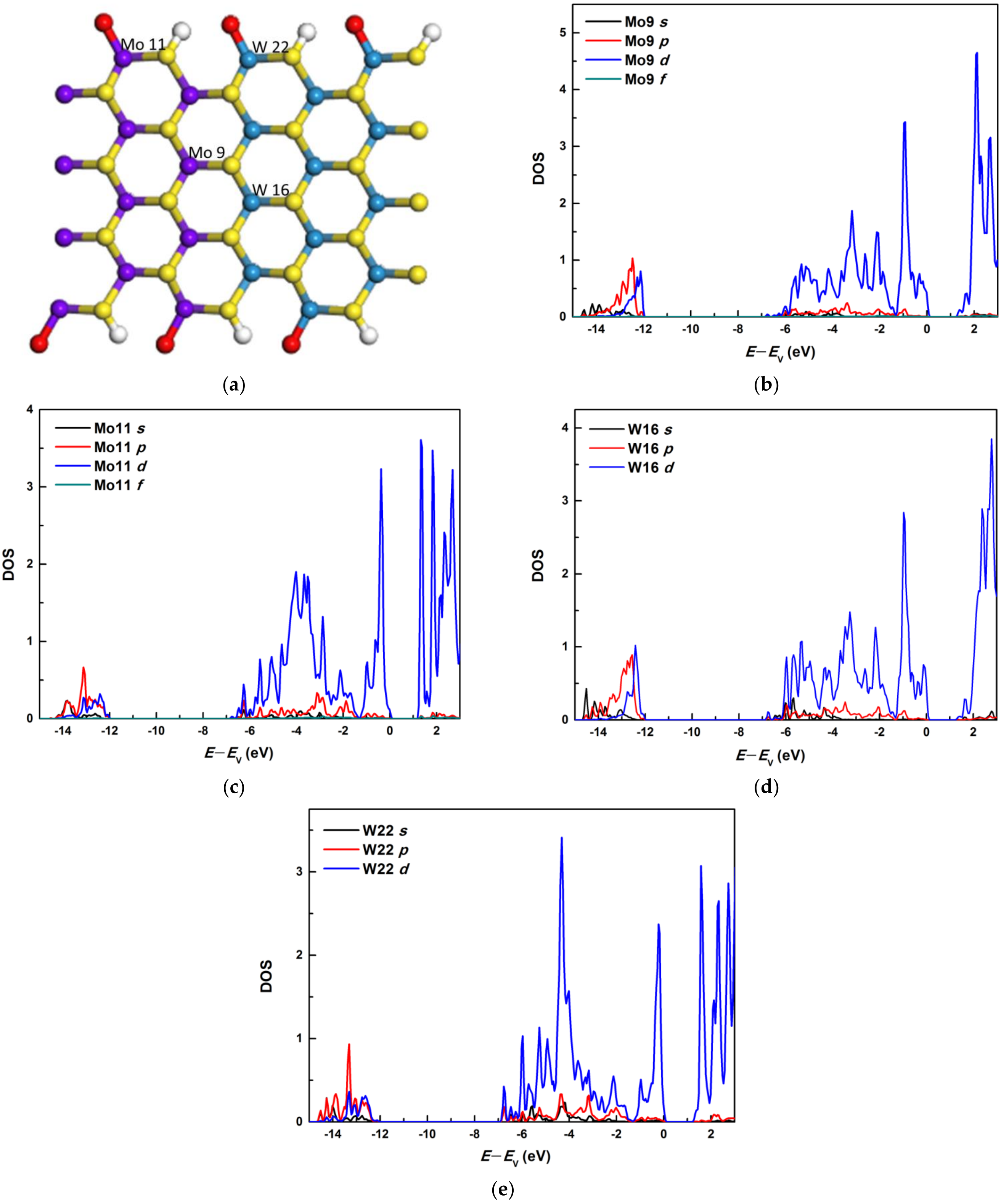

Figure S8. (**a**)The relaxed geometry of 8-ac-$(MoS_2)_3/(WS_2)_3$-NR-asym-H-O, edge and inside atoms for Mo and W are labeled. The partial density of states (pdos) for (**b**) inside Mo9 atom; (**c**) edge Mo11 atom; (**d**) inside W16 atom; (**e**) edge W22 atom of 8-ac-$(MoS_2)_3/(WS_2)_3$-NR-asym-H-O nanoribbon.

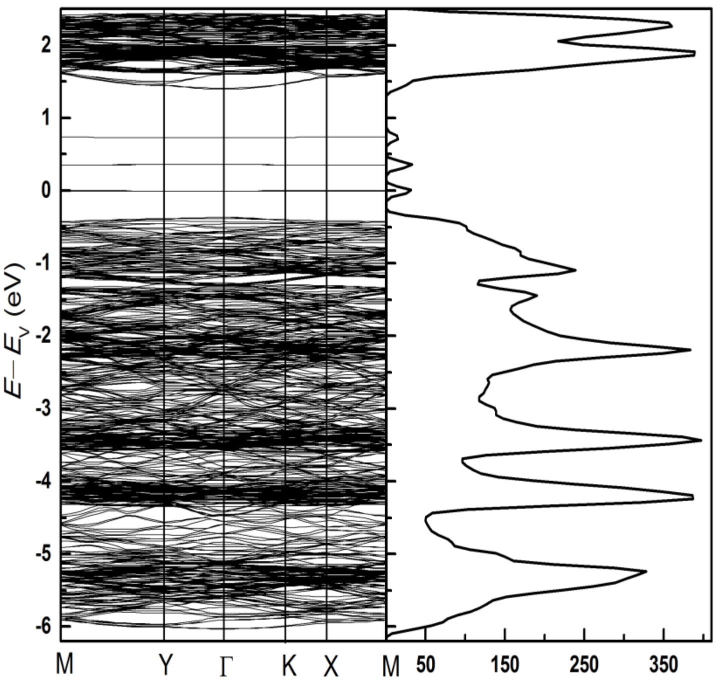

Figure S9. The band structure and dos of MoS_2 SL with a Mo vacancy in the middle.

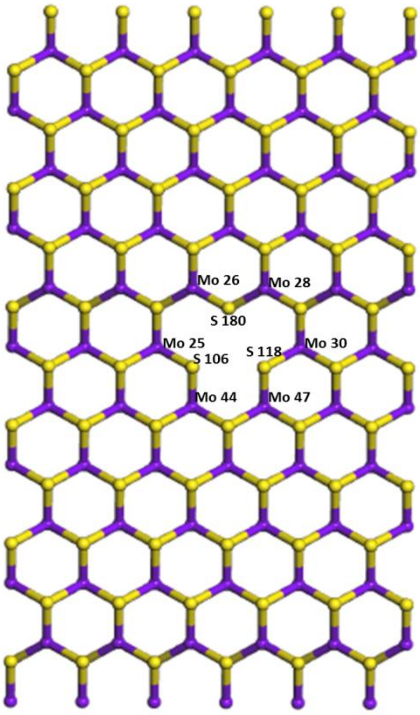
Mo 26
Mo 28
S 180
Mo 25
S 106
S 118
Mo 30
Mo 44
Mo 47

(**a**)

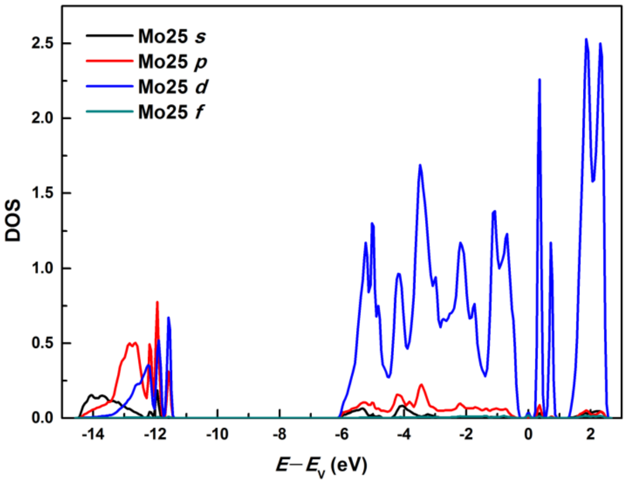
Mo25 s
Mo25 p
Mo25 d
Mo25 f
DOS
0.0
0.5
1.0
1.5
2.0
2.5
-14
-12
-10
-8
-6
-4
-2
0
2
E−E_V (eV)

(**b**)

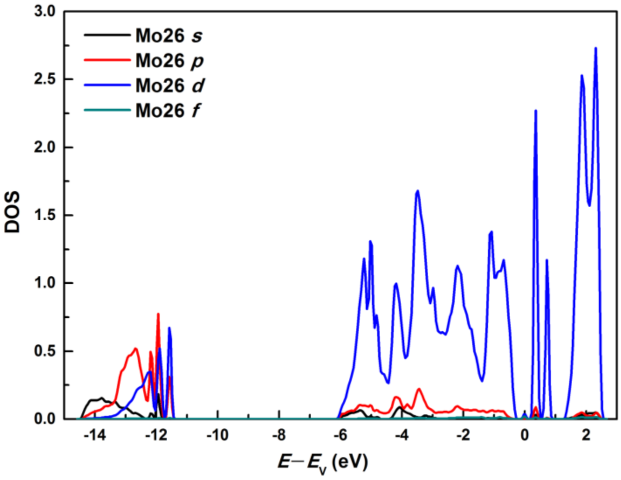
Mo26 s
Mo26 p
Mo26 d
Mo26 f
DOS
0.0
0.5
1.0
1.5
2.0
2.5
3.0
-14
-12
-10
-8
-6
-4
-2
0
2
E−E_V (eV)

(**c**)

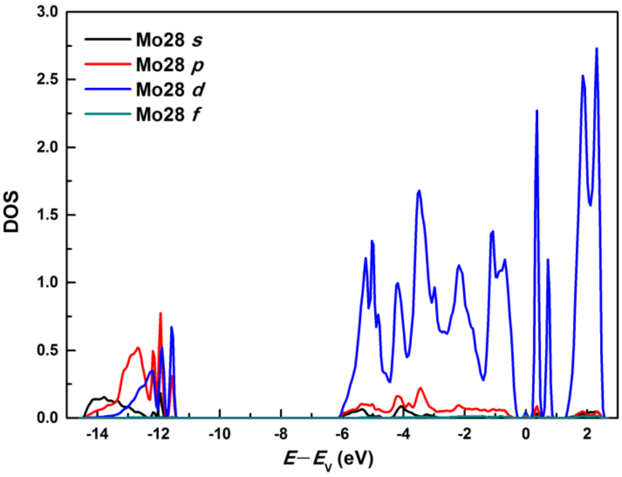
Mo28 s
Mo28 p
Mo28 d
Mo28 f
DOS
0.0
0.5
1.0
1.5
2.0
2.5
3.0
-14
-12
-10
-8
-6
-4
-2
0
2
E−E_V (eV)

(**d**)

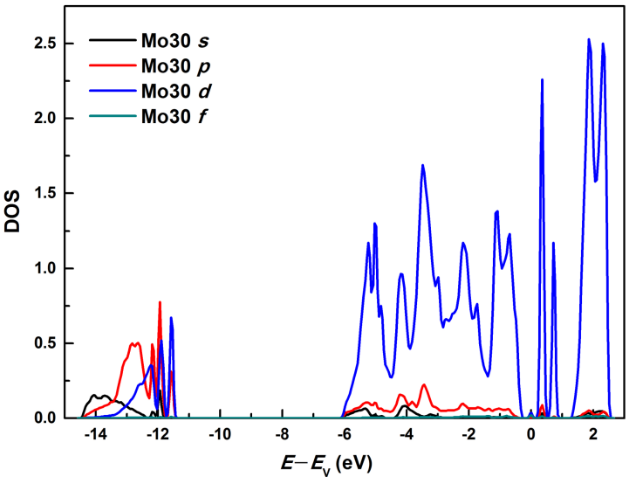
Mo30 s
Mo30 p
Mo30 d
Mo30 f
DOS
0.0
0.5
1.0
1.5
2.0
2.5
-14
-12
-10
-8
-6
-4
-2
0
2
E−E_V (eV)

(**e**)

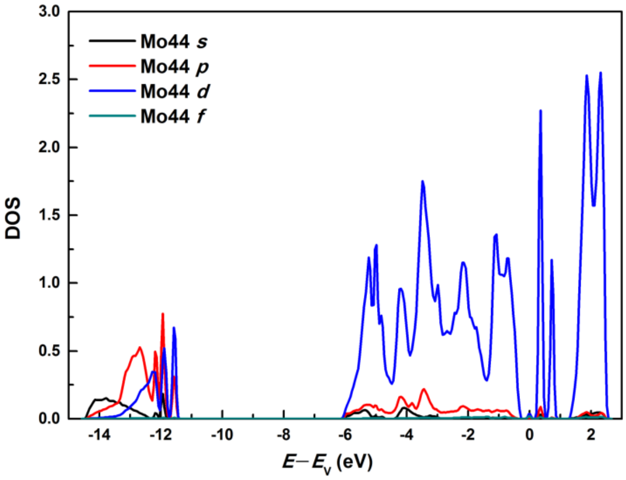
Mo44 s
Mo44 p
Mo44 d
Mo44 f
DOS
0.0
0.5
1.0
1.5
2.0
2.5
3.0
-14
-12
-10
-8
-6
-4
-2
0
2
E−E_V (eV)

(**f**)

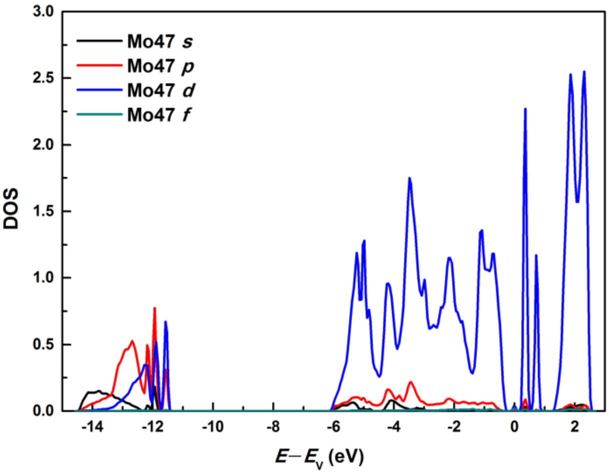

(g)

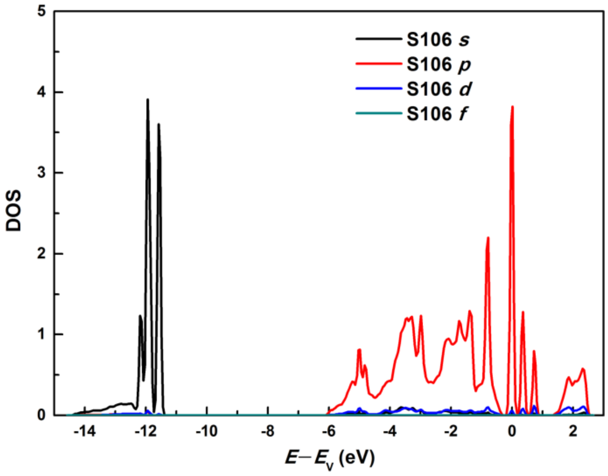

(h)

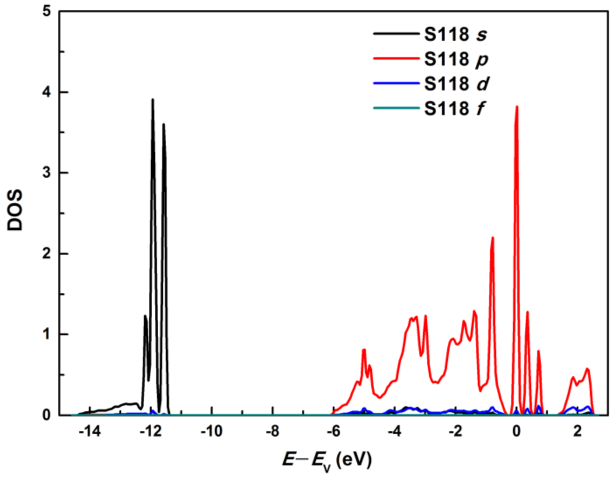

(i)

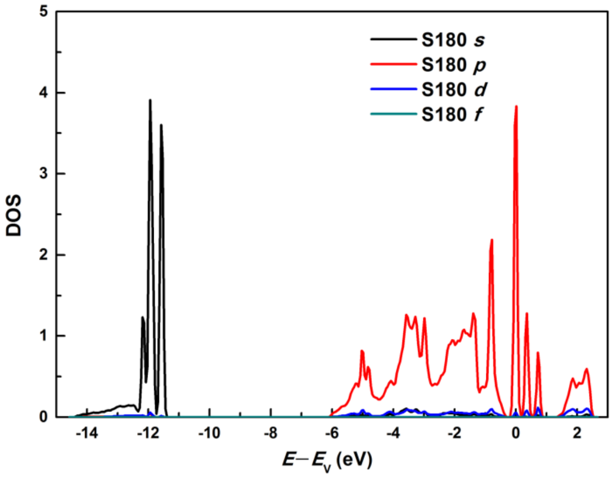

(j)

Figure S10. (**a**)The geometry of MoS_2 SL with a Mo vacancy in the middle and the atoms around Mo vacancy are labeled. The partial density of states (pdos) for atoms around Mo vacancy: (**b**) Mo25 atom; (**c**) Mo26 atom; (**d**) Mo28 atom; (**e**) Mo30 atom; (**f**) Mo44 atom; (**g**) Mo47 atom; (**h**) S106 atom; (**i**) S118 atom; (**j**) S180 atom of MoS_2 SL with a Mo vacancy in the middle.

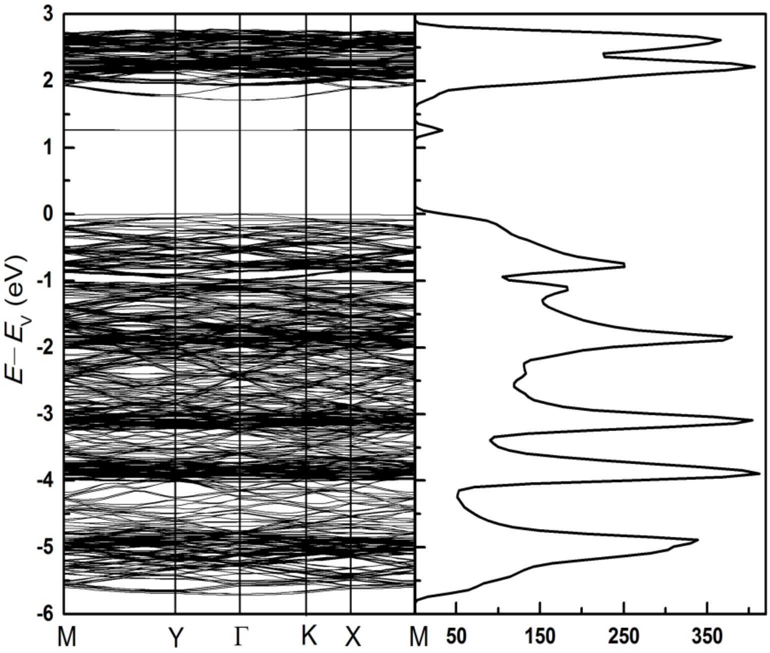

Figure S11. The band structure and dos of MoS_2 SL with a S vacancy in the middle.

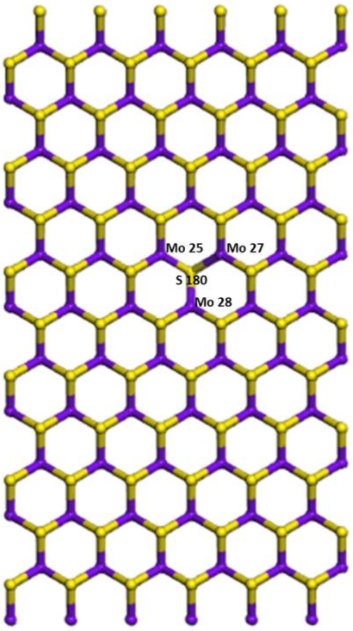

(**a**)

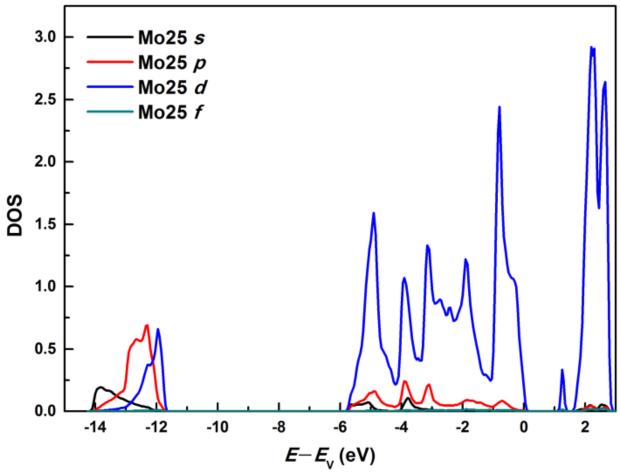

(**b**)

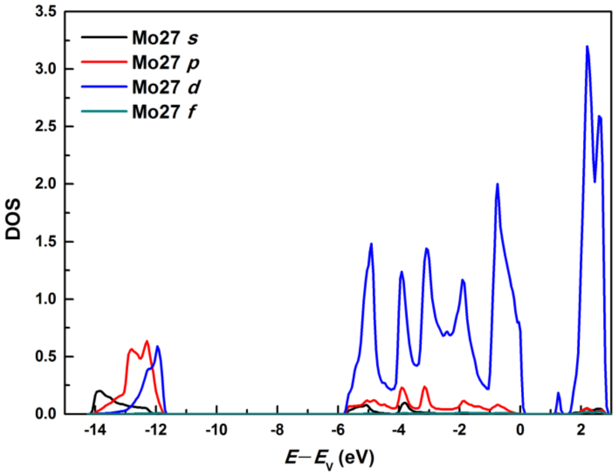

(**c**)

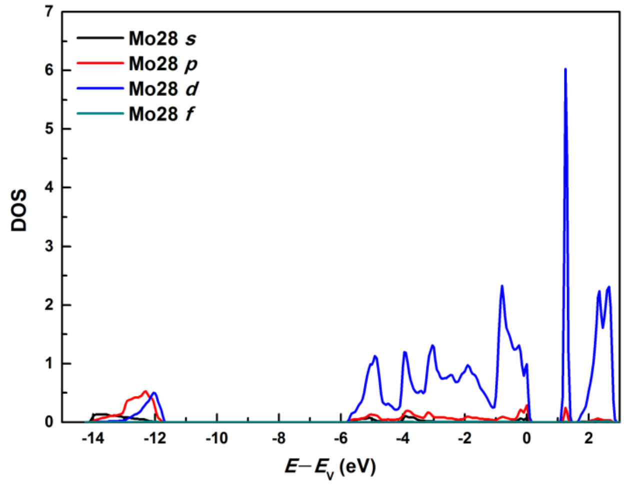

(**d**)

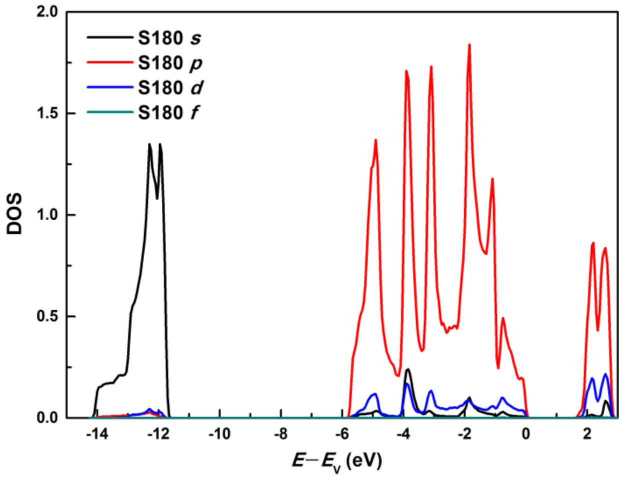

(**e**)

Figure S12. (**a**)The geometry of MoS_2 SL with a S vacancy in the middle and the atoms around S vacancy are labeled. The partial density of states (pdos) for atoms around S vacancy: (**b**) Mo25 atom; (**c**) Mo27 atom; (**d**) Mo28 atom; (**e**) S180 atom of MoS_2 SL with a S vacancy in the middle.
